# Semi-rational engineering an aldo–keto reductase for stereocomplementary reduction of α-keto amide compounds

**DOI:** 10.1186/s12934-023-02225-9

**Published:** 2023-10-15

**Authors:** Ruixuan Bai, Baoling Chen, Liangyu Zheng

**Affiliations:** Key Laboratory for Molecular Enzymology and Engineering of Ministry of Education, School of Life Sciences, Jilin University, Changchun, 130012 China

**Keywords:** α-keto amides, α-hydroxyl amides, Aldo–keto reductase, Stereocomplementary, Semi-rational engineering, Switch

## Abstract

Enantio-pure α-hydroxy amides are valuable intermediates for the synthesis of chiral pharmaceuticals. The asymmetric reduction of α-keto amides to generate chiral α-hydroxy amides is a difficult and challenging task in biocatalysis. In this study, iolS, an aldo–keto reductase from *Bacillus subtilis 168* was exhibited as a potential biocatalyst, which could catalyze the reduction of diaryl α-keto amide such as 2-oxo-*N*, 2-diphenyl-acetamide (ONDPA) with moderate *S*-selectivity (76.1%, *ee*) and 60.5% conversion. Through semi-rational engineering, two stereocomplementary variants (I57F/F126L and N21A/F126A) were obtained with *ee* value of 97.6% (*S*) and 99.9% (*R*) toward ONDPA (**1a**), respectively, delivering chiral α-hydroxy amide with > 98% conversions. Moreover, the excellent *S*- and *R*-preference variants displayed improved stereoselectivities toward the other α-keto amide compounds. Molecular dynamic and docking analysis revealed that the two key residues at 21 and 126 were identified as the “switch”, which specifically controlled the stereopreference of iolS by regulating the shape of substrate binding pocket as well as the substrate orientation. Our results offer an effective strategy to obtain α-hydroxy amides with high optical purity and provide structural insights into altering the stereoselectivity of AKRs.

**Graphical Abstract:**

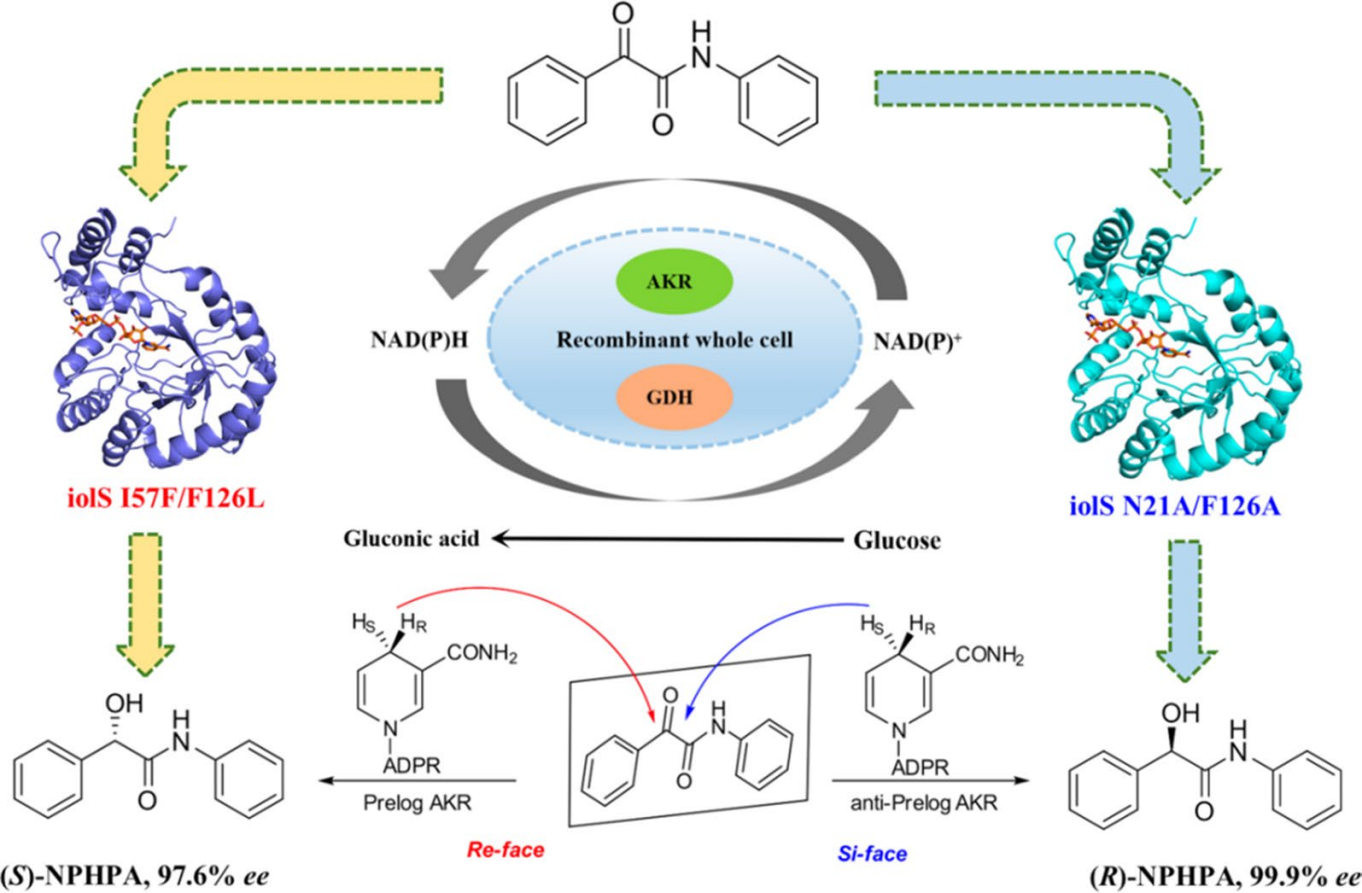

**Supplementary Information:**

The online version contains supplementary material available at 10.1186/s12934-023-02225-9.

## Introduction

Optically pure α-hydroxy amides are very valuable structural motifs for biologically active compounds such as anticonvulsant, bradykinin antagonists and antibiotics [1, 2]. In addition, these candidates also serve as important intermediates in the field of synthetic chemistry due to their ability to be converted into a variety of functionalized compounds [3, 4]. This has stimulated numerous efforts to find new ways to produce α-hydroxy amides with optical activity. The most commonly used chemical methods for synthesizing chiral α-hydroxy amides are enantioselective Passerini-type reaction [5], asymmetric ring opening of α, β-epoxy amides [6], oxidation kinetic resolution of racemic α-hydroxy amides [7] and asymmetric reduction of α-keto amides [8]. Among the various methods, the enantioselective reduction of α-keto amides provides a direct way to obtain these chiral compounds. However, in α-keto amides, the benzoyl carbonyl is sandwiched between the carbonyl carbon of the amide and aromatic carbon, both with sp [2] character, and obtaining high enantioselectivity in this class of molecules is a challenging task [9]. Recently, studies have reported the asymmetric reduction of α-keto amides by chiral metal complexes. The RuCl [(*R*, *R*)-Teth-TsDPEN]-catalyzed reduction of 2-oxo-*N*, 2-diphenyl-acetamide (ONDPA) can reach 98% yield and the enantiomeric excess (*ee*) value of the (*S*)-2-hydroxy-*N*, 2-diphenylacetamide is 44% [10]. CuF, (*S*)-DTBM-SEGPHOS, and (EtO)_3_SiH are used as chemical catalysts for enantioselective reduction of α-keto amides to achieve (*R*)-hydroxy amides with 97% yield and 99% *ee* [11]. However, the usage of metal catalysts is limited due to strict regulatory on the heavy metal and high costs.

Biocatalysts have natural advantages such as environmental friendliness, high enantioselectivity, and gentle reaction conditions compared to metal catalysts [12]. Although bio-reduction of carbonyl compounds is ubiquitous, only a few examples of biocatalytic asymmetric reduction toward α-keto amides have been described. The carbonyl reductase IF0 0708 purified from *Candida parapsilosis* can reduce *N*-methyl derivatives of indoline-2,3-diketone to (*R*)-hydroxy amide with 28% yield and 99% *ee* [13]. The whole cell of *Candida parapsilosis* ATCC730 has been reported to asymmetrically reduce α-keto amide compounds to *R*-alcohol with a lower *ee* value (12–94%) [14]. Obviously, the undesirable stereoselectivity and activity of reported biocatalysts could not satisfy the demand for the synthesis of pharmaceutical intermediates. Thus, it is particularly vital to find advantageous enzymes for the asymmetric synthesis of chiral α-hydroxy amides.

**Scheme 1:**
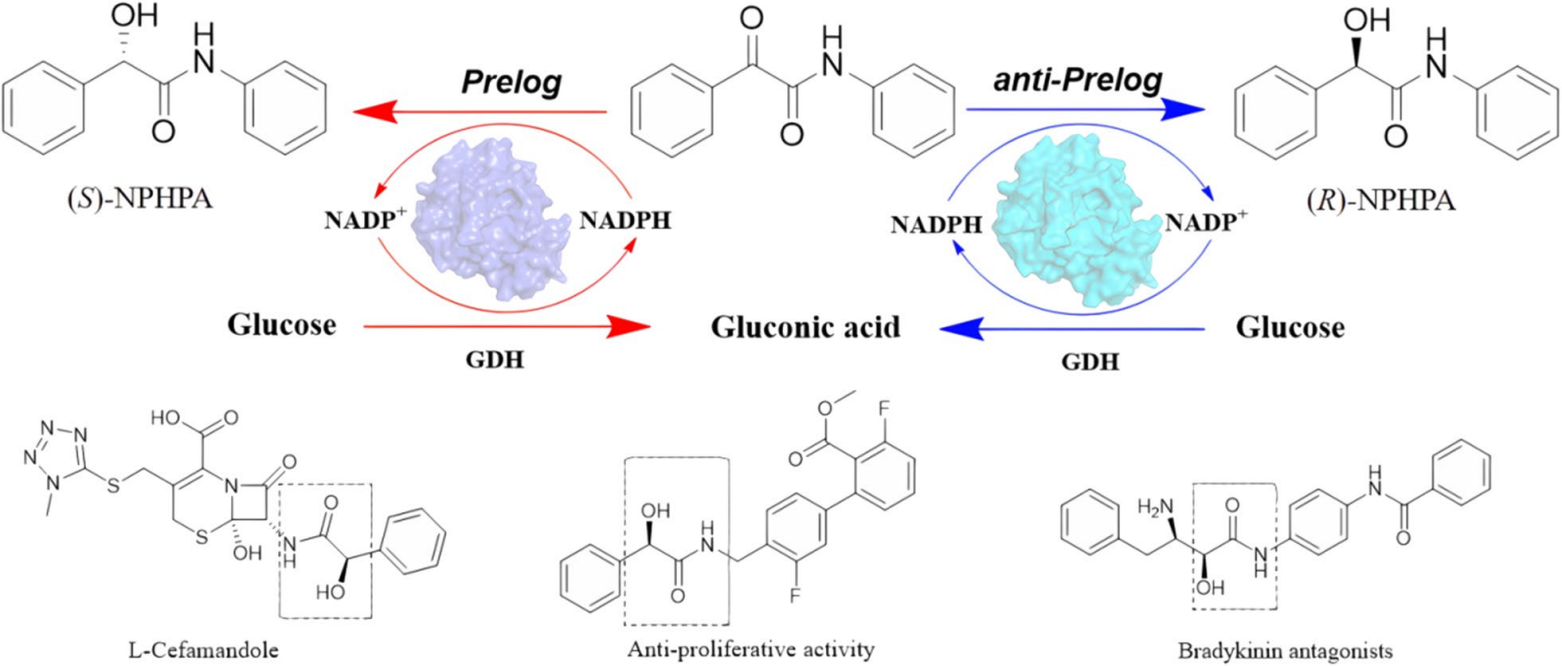
Asymmetric reduction of 2-oxo*-N*, 2-diphenyl-acetamide (ONDPA) into (*S*)- and (*R*)-* N*-phenyl-2-hydroxy-2-phenylacetamide (NPHPA) by iolS variants and the related drugs with NPHPA as a key intermediate

It is well known that the potential value of aldo–keto reductase (AKR) as a biocatalyst cannot be underestimated. The AKR superfamily is currently composed of over 190 proteins, sharing the (α/β)_8_ barrel structure and the conserved catalytic tetrad Asp-Tyr-Lys-His [15–17]. In addition, these NAD(P)H-dependent enzymes can catalyze the reduction of prochiral ketones with high chemoselectivity, regioselectivity, and stereoselectivity [18], providing a valuable pathway for the synthesis of chiral alcohols. Most of the asymmetric reduction of prochiral ketones catalyzed by AKRs comply with Prelog’s rule [19]. AKRs with excellent anti-Prelog stereoselectivity are scarce in nature [20]. However, some anti-Prelog chiral alcohols could display crucial function in pharmaceutical synthesis. For instance, (*R*)-α-hydroxy amides are more effective than (*S*)-α-hydroxy amides as the key building blocks for synthesizing anti-proliferative activity and L-Cefamandole [21]. Therefore, the development of stereocomplementary AKR is of vital importance to elucidate the mechanism of stereochemistry and synthesis of chiral α-hydroxy amides. (Scheme 1).

In this study, an aldo–keto reductase (iolS) [22] from *Bacillus subtilis 168* was cloned and expressed in *E. coli* BL21(DE3). The wild-type (WT) iolS exhibited a moderate enantioselectivity and conversion in the reduction of ONDPA (**1a**). Through semi-rational engineering, two stereocomplementary variants with enhanced activities and stereoselectivities were obtained, which could be used to prepare a variety of (*S*)- and (*R*)-α-hydroxy amides. The stereoselective improvement and inversion of iolS is explained by theoretical calculation based on molecular dynamics (MD) simulation.

## Results and discussion

### Identification of key amino acid residues

In the preliminary experiment, two AKRs (iolS and yhdN) were screened using ONDPA (**1a**) as a substrate. To solve the problem of adding extra cofactors, the recombinant AKRs-GDH whole-cell were constructed and employed for the asymmetric reduction of **1a**. While optimizing the catalytic reaction, it was found that the constructed recombinant whole cells exhibited better catalytic effects than pure enzyme. They not only greatly reduced the reaction time to 3 h, but the whole-cell iolS-GDH and yhdN-GDH also improved the conversions to 60.5% and 48.4%, respectively. Among the two AKRs, the asymmetric reduction of ONDPA catalyzed by iolS-GDH exhibited a higher stereoselectivity (76.1%, *S*) (Additional file 1: Table S2). Thus, iolS was selected for further research.

**Fig. 1:**
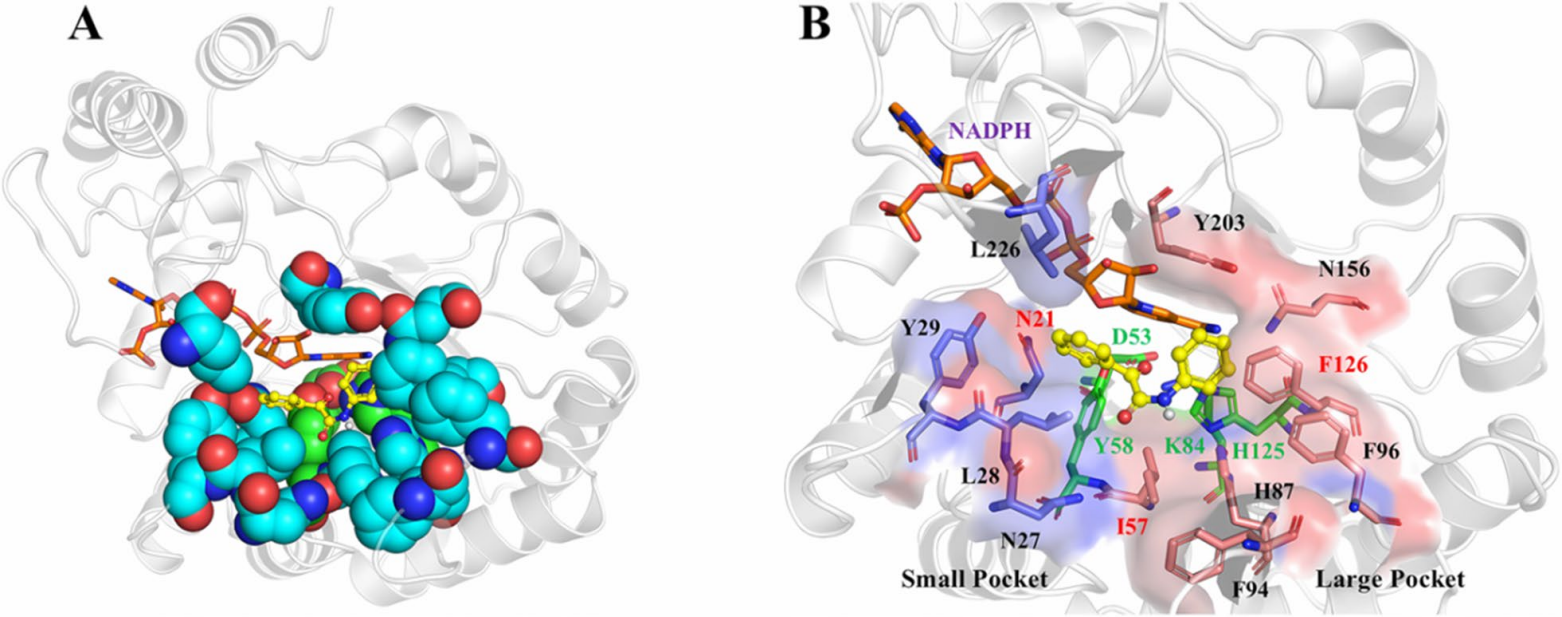
Docking of ONDPA into the substrate binding pocket of iolS. **A** The spatial shape of the substrate binding pocket of WT, **B** illustration of the key residues at the small and large binding pockets. Residues in the large and small binding pockets are depicted in pink and slate, respectively. Orange: NADPH; green: catalytic D53, Y58, K84, and H125; yellow: ONDPA

To improve the catalytic performances of iolS, molecular docking was performed between iolS and ONDPA to identify potential amino acid residues that might affect its activity and stereoselectivity. In accordance with the diagram of the substrate binding pocket in Fig. 1, the carbonyl oxygen pointed to the catalytic residue Tyr58, and the carbonyl C atom was in a favorable state for hydride transfer from NADPH. As the potentially beneficial residues that are responsible for controlling enzyme stereoselectivity are often situated in or near the substrate binding pocket, the 12 pocket-decorating amino acid residues including five (N21, N27, L28, Y29, and L226) and seven (I57, H87, F94, F96, F126, N156, and Y203) in the small and large binding pocket, respectively, were identified (Fig. 1B).

To clarify the role of each selected amino acid residue on the activity and enantioselectivity of iolS, alanine scanning was then performed [23]. As shown in Table 1, the variants N27A, F94A, F96A, N156A, Y203A, and L226A exhibited similar selectivity and catalysis efficiency in comparison with WT, which suggested that the six amino acid sites were not the key residues. It was observed that the variants N21A, L28A, Y29A, I57A, H87A, and F126A caused substantial changes in *ee* values of the product. In particular, the variants N21A (93.2%,* R*), L28A (6.38%, *R*), and Y29A (4.2%, *R*) displayed the opposite selectivity in the reduction of **1a**. This indicated that the expanded space of the small binding pocket can affect the stereoselectivity recognition between enzyme and substrate. The alanine mutants I57A (64.5%, *S*) and H87A (1.5%, *S*) situated in the large pocket exhibited lower enantioselectivity than that of WT (76.1%, *S*), and F126A showed inverted selectivity (15.1%, *R*), which revealed that I57, H87 and F126 were vital sites in the large binding pocket. In view of this, the six amino acid residues, namely N21, L28, Y29, I57, H87, and F126 were deemed to be the potential hotspots that needed to be further studied. Large amounts of variants were then obtained by using site-directed saturation mutagenesis technology, and their catalytic abilities towards the asymmetric reduction of ONDPA were also evaluated.

**Table 1:** Alanine scanning of iolS for identifying the hotspots^*a*^

Variants	*ee* (%)^*b*^	*Conv.* (%)^*c*^	Favored enantiomer
N21A	93.2 ± 0.5	97.3 ± 0.6	*R*
N27A	74.1 ± 0.3	56.4 ± 0.4	*S*
L28A	6.3 ± 0.2	43.6 ± 0.3	*R*
Y29A	4.2 ± 0.3	48.3 ± 0.4	*R*
I57A	64.5 ± 0.4	58.5 ± 0.7	*S*
H87A	1.5 ± 0.1	39.8 ± 0.4	*S*
F94A	71.3 ± 0.5	61.8 ± 0.2	*S*
F96A	75.2 ± 0.4	58.7 ± 0.6	*S*
F126A	15.1 ± 0.2	50.2 ± 0.4	*R*
N156A	76.7 ± 0.5	62.4 ± 0.5	*S*
Y203A	70.1 ± 0.4	54.2 ± 0.4	*S*
L226A	72.7 ± 0.3	64.4 ± 0.7	*S*

^a^Reaction condition: 4 mM substrate **1a**, 200 mM glucose and 0.1 g wet whole-cell in potassium phosphate buffer (100 mM, pH 7.0, 500 μL) at 30 °C. Experiments were conducted in duplicate

^b^The ee values were measured by chiral HPLC; the absolute configuration was determined by comparing the retention times with literature data

^c^Conversions were determined by chiral HPLC analysis

### Saturation mutagenesis of key residues

**Fig. 2:**
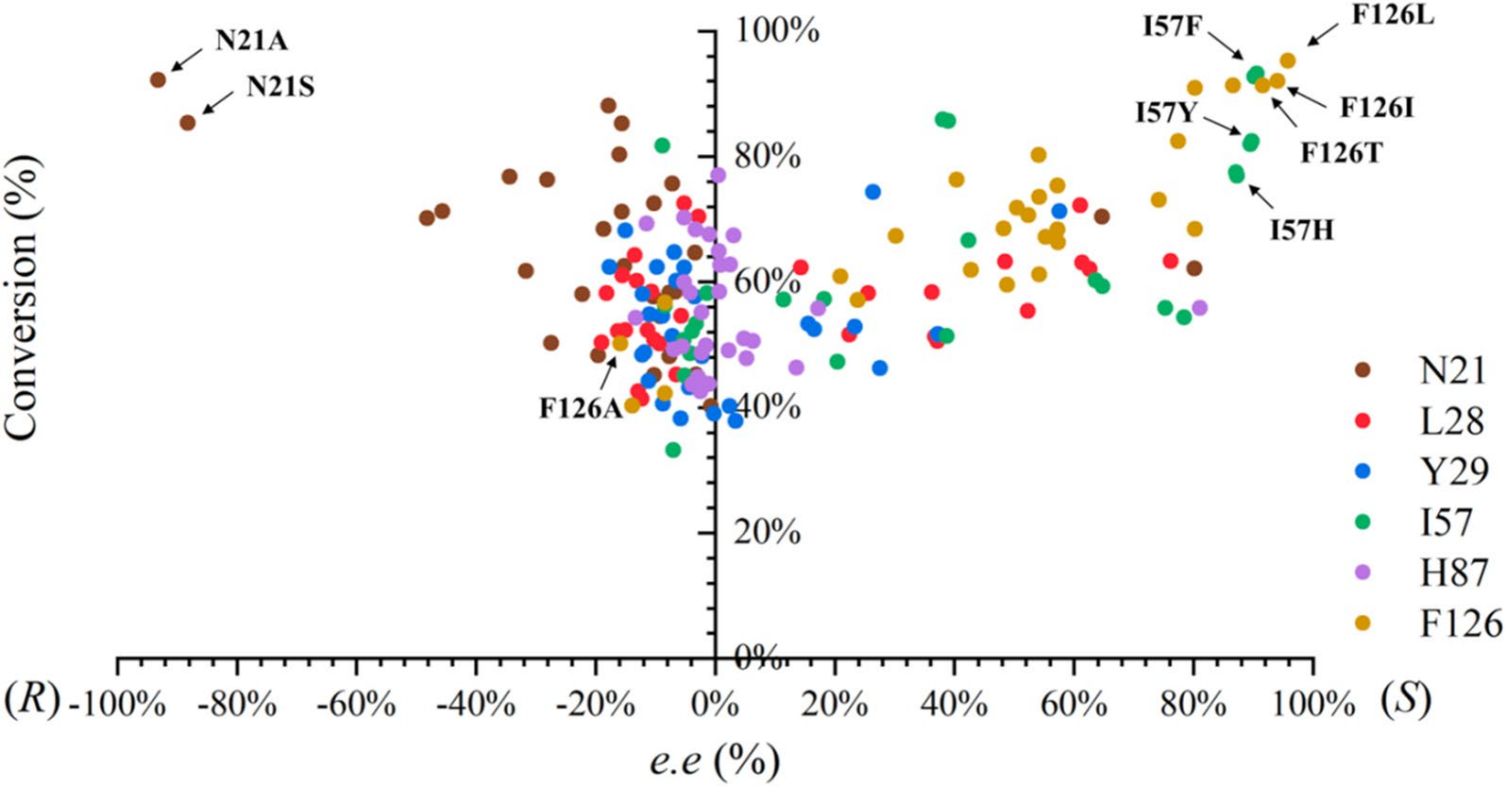
The effect of single mutants on stereoselectivity and conversion in the reduction of ONDPA

The NNK codon was used for site-directed saturation mutagenesis (SM) (Fig. 2). The stereoselectivity and conversion of the sequenced variants have been listed in Additional file 1: Table S3. For residue 21, no mutants were conducive to improving the *ee* value of *S*-NPHPA. Most mutants displayed *R*-selectivity preference toward ONDPA (**1a**). Sequencing results showed that variants N21A and N21S catalyzed the reduction of ONDPA to *R*-NPHPA with *ee* values of 93.2% and 88.2%, respectively. Moreover, the conversion of both mutants was above 93%, which was significantly higher than that of WT. As shown in Additional file 1: Fig. S1A, the enlarged space of the small binding pocket can accommodate the *N*-phenylformamide group of the ONDPA well. These results suggested that the residues at site 21 are critical for substrate localization and identification.

For residue 57, the stereoselectivities of I57F (90.6%, *S*), I57Y (89.9%, *S*), and I57H (87.3%, *S*) were higher than that of WT. In particular, the variant I57F demonstrated the highest conversion of 93.2%. The analysis of receptor-ligand interaction showed that the mutation I57F formed a new π–π interaction with the *N*-phenylmethylamide group of ONDPA, which could fix the large substituents on the binding pocket and be conducive to the generation of the dominant configuration (Additional file 1: Fig. S1B). This suggested that the mutation of amino acid residues into side chains with aromatic or heterocyclic elements might contribute to substrate orientation and regulate the binding position of the substrate to the enzyme, thus controlling the stereochemistry of the reaction.

The phenylalanine at site 126 was located on the β-fold in the substrate-binding domain of iolS, and beneficial mutants, F126L (95.3% *ee*, *S*), F126I (94.0% *ee*, *S*), and F126T (91.5% *ee*, *S*) with conversions above 90% were obtained. The variants F126G (8.4% *ee*, *R*) and F126S (13.9% *ee*, *R*) reversed the stereopreference when the small amino acid was introduced in site 126. This indicated that the stereoselectivity of iolS could be influenced by steric hindrance effects at this site. According to the Additional file 1: Fig. S1C, the smaller side chain of F126L was conducive to the enlarged space of the large binding pocket and formed a new π–σ interaction between the *N*-phenylformamide group and L126, which improved the binding affinity between ONDPA and iolS. This could account for the increased activity and stereoselectivity. When F126 was replaced by the alanine, the π–π interaction between 126 site and *N-*phenylformamide groups disappeared. This might improve the steric flexibility in binding of ONDPA and consequently changed the binding mode of substrate (Additional file 1: Fig. S1D). Furthermore, variants with decreased or reversed stereoselectivity were discovered in saturation mutants at the Y28, L29, and H87 sites compared to WT. Although this was influential, the results were not ideal. Therefore, these variants are not the primary focus of this study.

### Promotion of enantioselectivity through combinatorial mutagenesis

**Fig. 3:**
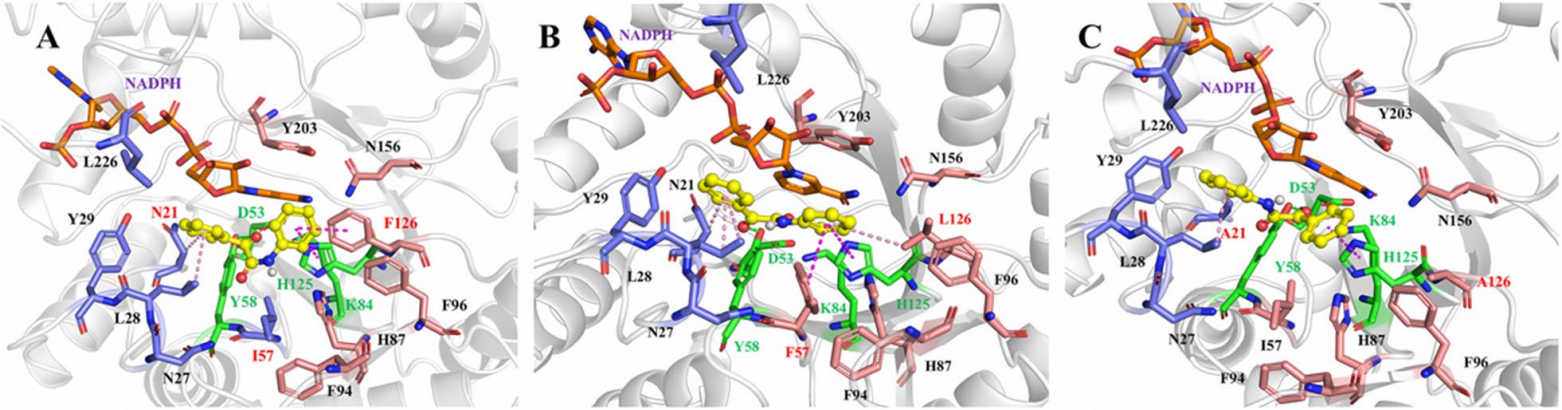
Analysis of substrate binding pockets and interactions between ligands and proteins of iolS and variants. Binding modes of pro-*S* conformation to WT iolS (**A**) and variant I57F/F126L (**B**); Binding modes of pro-*R* conformation to variant N21A/F126A (**C**). Yellow: ONDPA; orange: NADPH; residues of binding pockets are shown by sticks; green: hydrogen bonds; magenta: π − π interactions; pink: π − σ and π − Alkyl interactions

Combinatorial mutagenesis was employed to achieve NPHPA (1b) with the appropriate *S*- and *R*-configurations (Table 2). All single point variants were classified into two groups, the variants with high *S*-selectivity were included in one group, such as F126L, F126I, I57F, I57H, and I57Y, and the other group contained the variants with inverted selectivity such as N21A, N21S, and F126A. Then, double mutants were rationally generated by combinatorial mutagenesis. The interaction analysis of iolS and its excellent variants with substrate **1a** is shown in Fig. 3. Comparison of other variants with* S*-preference, I57F/F126L showed the highest conversion (98.5%) and stereoselectivity (97.6%, *S*). The introduction of I57F and F126L resulted in the *N*-phenylmethylamide group forming π–π and π–σ interactions with Phe57 and Leu126, respectively (Fig. 3B). According to docking results, the variant I57F/F126L could enhance the binding affinity with the phenyl group of ONDPA (**1a**) in the large binding pocket. As substrate **1a** is a hydrophobic compound with two aromatic rings, it was conjectured that the enhanced hydrophobic effects can contribute to the improvement of catalytic efficiency and stereoselectivity.

**Table 2:** Enantioselectivity and conversion of iolS and double mutants toward ONDPA^*a*^

Variants	*ee* (%)^*b*^	*Conv*. (%)	Favored enantiomer
WT	76.1 ± 0.6	60.5 ± 0.5	*S*
I57F/F126I	94.3 ± 0.7	98.4 ± 0.8	*S*
I57Y/F126I	92.6 ± 1.3	95.3 ± 0.6	*S*
I57H/F126I	90.0 ± 0.4	83.0 ± 0.5	*S*
I57F/F126L	97.6 ± 0.4	98.5 ± 0.6	*S*
I57Y/F126L	94.2 ± 0.5	96.4 ± 0.4	*S*
I57H/F126L	93.7 ± 0.6	93.2 ± 0.4	*S*
N21A/F126A	99.9 ± 0.1	99.9 ± 0.2	*R*
N21S/F126A	99.9 ± 0.1	98.2 ± 0.4	*R*

^a^Reaction condition: 4 mM substrate **1a**, 200 mM glucose and 0.1 g wet whole-cell in potassium phosphate buffer (100 mM, pH 7.0, 500 μL) at 30 °C. Experiments were conducted in duplicate

^b^The ee values were measured by chiral HPLC; the absolute configuration was determined by comparing the retention times with literature data

^c^Conversions were determined by chiral HPLC analysis

For double variants with *R*-preference, both the variants N21A/F126A and N21S/F126A, displayed an *ee* value of 99.9%. Remarkably, N21A/F126A possessed a higher conversion of 99.9%, indicating that the reduction of the steric hindrance near the active site is a key factor to improve the catalytic activity of iolS. Based on the N21A, the substrate binding pocket was further enlarged by introducing F126A, which led to *N*-phenylmethylamide group rotation to the opposite direction (Fig. 3C). In this binding model, the binding poses of substrates in the variants N21A/F126A were completely distinct from those in the iolS. The residues A21 and L28 formed two new π–σ interactions with the *N*-phenylformamide group which enhanced the binding of bulky substituents to small binding pockets. The anti-Prelog conformations were also stabilized by π–π interaction between the phenyl group and the imidazole group of H125. In summary, under the guidance of semi-rational design, double mutants I57F/F126L and N21A/F126A were constructed with high stereoselectivity and conversion in the synthesis of (*S*)-NPHPA and (*R*)-NPHPA, respectively. In addition, residues at 21 and 126 were found to play a crucial role in controlling the switch between the Prelog and anti-Prelog of iolS.

### Kinetic parameter analysis of iolS and variants

The kinetics of WT iolS and beneficial variants were determined. Nonlinear fitting was used to calculate the *K*_m_ and *k*_cat_ values. The catalytic efficiencies (*k*_cat_/*K*_m_) of variants toward ONDPA are presented in Table 3. The *K*_m_ and *k*_cat_ values of iolS were 1.81 mM and 3.53 s^−1^, respectively. In the variants of *S*-selectivity, the variant F126L showed the lowest *K*_m_ of 1.55 mM and displayed improved binding affinity toward ONDPA (**1a**). The best variant I57F/F126L exhibited the highest *k*_cat_ value of 4.32 s^−1^ and *k*_cat_/*K*_m_ value of 2.51 s^−1^·mM^−1^, which are higher than those of the WT and single variants. As for variants of *R*-selectivity, the *K*_m_ value of N21A was 1.16 mM, the highest binding affinity among all the variants including *S*-selective variants. Remarkably, the *k*_cat_ and *k*_cat_/*K*_m_ of variant N21A/F126A were increased to 6.40 s^−1^ and 3.88 s^−1^·mM^−1^, respectively, and an approximate twofold enhanced *k*_cat_/*K*_m_ value compared with WT. This indicated that the double variant N21A/F126A greatly improved the catalytic efficiency of the reaction and achieved the highest conversion toward ONDPA. Moreover, the *k*_cat_ value increase with *R*-selective variants was more considerable than that for the variants with *S*-preference. These results indicated that the substitutions at N21, I57, and F126 of iolS could affect both the enantioselectivity and catalytic efficiency of the asymmetric reduction reaction toward ONDPA.

**Table 3:** Kinetic Parameters of iolS and variants toward ONDPA^*a*^

Enzyme	*K*_m_ (mM)	*k*_cat_ (s^−1^)	*k*_cat_ /*K*_m_ (s^−1^·mM^−1^)
iolS WT	1.81 ± 0.13	3.53 ± 0.12	1.95
I57F	1.72 ± 0.13	3.96 ± 0.23	2.32
F126L	1.55 ± 0.22	3.81 ± 0.13	2.45
N21A	1.16 ± 0.16	4.10 ± 0.12	3.53
I57F/F126L	1.71 ± 0.11	4.32 ± 0.14	2.51
N21A/F126A	1.65 ± 0.12	6.40 ± 0.25	3.88

^a^Nonlinear regression of the kinetic data are shown in Additional file 1: Fig. S2

### Molecular dynamic simulation in the prereaction state

The prereaction state displayed in the computational simulation may reflect the initial stereoselective recognition of the enzyme [24]. According to the catalytic reaction mechanism of aldo–keto reductase, His125 stabilizes the substrate, Tyr58 functions as the catalytic center, and Asp53 and Lys84 reduce the p*K*_a_ of the hydroxyl group of Tyr58 to promote the protonation. The reaction begins with the transfer of protons from Tyr58-OH to the carbonyl oxygen atom of ONDPA [25]. The carbonyl of ONDPA is subsequently changed into hydroxyl by the transfer of hydrogen atoms of NADPH to the carbonyl carbon. Therefore, the distance between carbonyl oxygen of ONDPA and Tyr58-OH, designated *d* (O_sub_ − OH_Y58_), and the distance between the carbonyl carbon of ONDPA and the hydrogen atom at C4 of NADPH, called *d* (C_sub_ − H4_NADPH_) could be used to assess whether the pre-reaction state is achieved [26]. Based on the previous studies on proton and hydride transfer [27], the conformational ratio of the distance between *d* (O_sub_ − OH_Y58_) ≤ 3.4 Å and *d* (C_sub_ − H4_NADPH_) ≤ 4.5 Å was used to calculate the probability of pre-reaction state formation. In addition, according to the configuration of ONDPA, the substrate in the prereaction state of the enzyme–substrate complex could be characterized as ONDPA_pro*S*_ and ONDPA_pro*R*_.

**Fig. 4:**
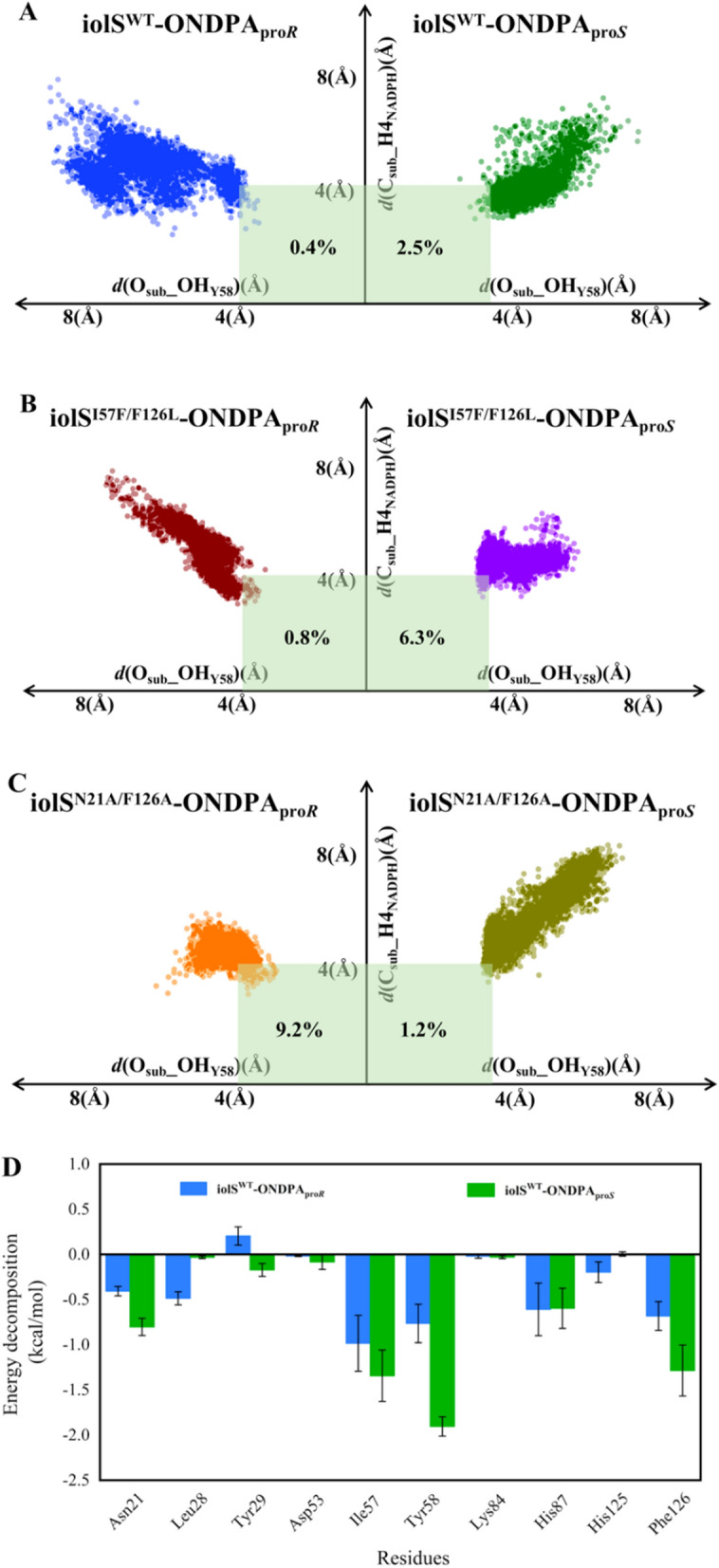

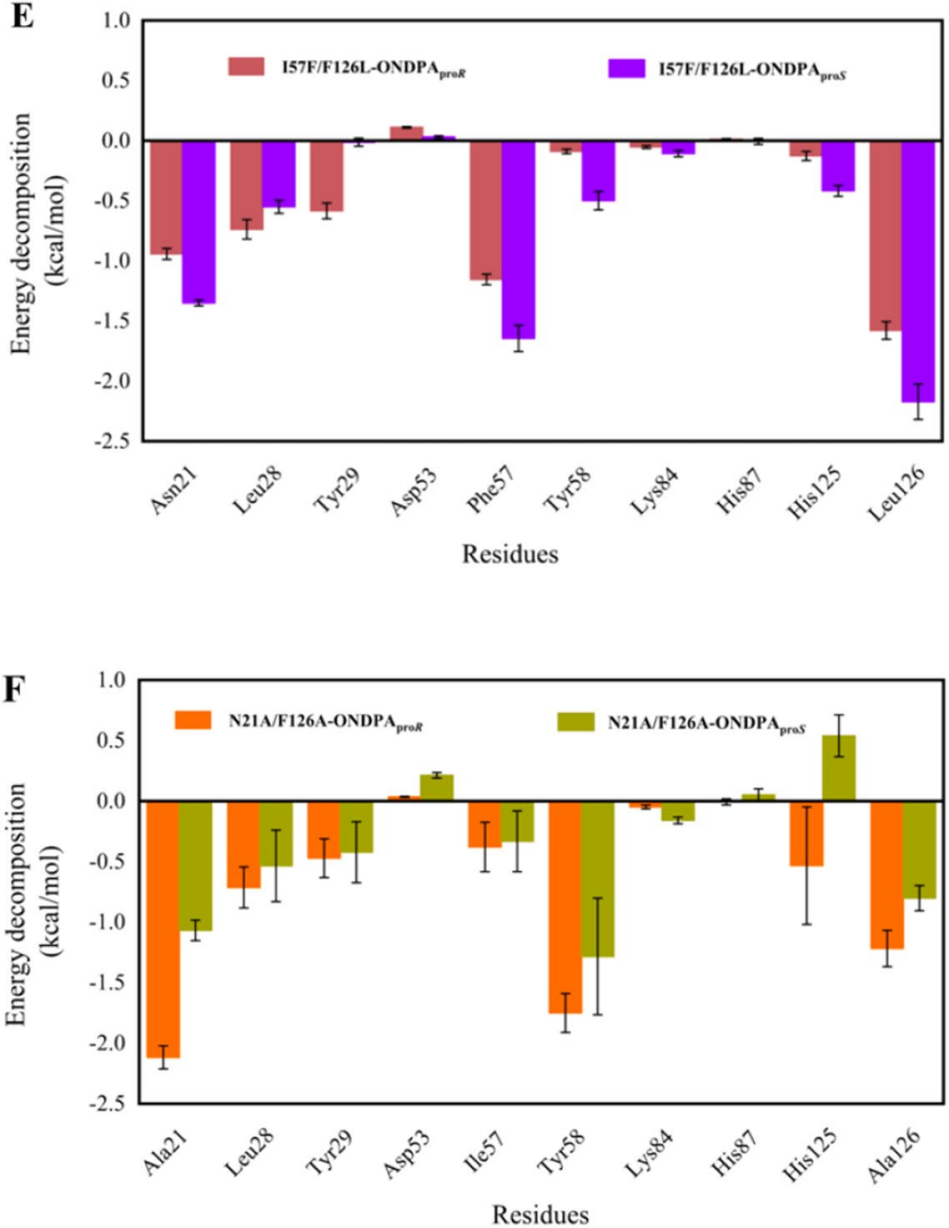
Conformation maps and energy decomposition of the residues in molecular dynamics simulations. **A** iolS-ONDPA_pro*R*_ (blue) and iolS-ONDPA_pro*S*_ (green); **B** I57F/F126L-ONDPA_pro*R*_ (dark red) and I57F/F126L-ONDPA_pro*S*_ (purple); **C** N21A/F126A-ONDPA_pro*R*_ (orange) and N21A/F126A-ONDPA_pro*S*_ (olive); energy contributions of key residues in the binding pocket to binding of ONDPA_pro*R*_ in iolS^WT^ (blue) and ONDPA_pro*S*_ in iolS^WT^ (green) (**D**), ONDPA_pro*R*_ in variant I57F/F126L (dark red) and ONDPA_pro*S*_ in I57F/F126L (purple) (**E**), ONDPA_pro*R*_ in variant N21A/F126A (orange) and ONDPA_pro*S*_ in N21A/F126A (olive) (**F**). The limits of “catalytic distances,” namely *d* (O_sub_ − OH_Y58_) ≤ 3.4 Å and *d* (C_sub_ − H4_NADPH_) ≤ 4.5 Å, in panels **A** − **C** are colored by green

As shown in Fig. 4, the orientation of ONDPA_pro*S*_ was observed in the stable prereaction state of iolS and variant I57F/F126L, while the pro-*R* conformation of ONDPA displayed higher probability of prereaction state of N21A/F126A. For the prereaction state of iolS^WT^-ONDPA_pro*S*_, I57F/F126L-ONDPA_pro*S*_ and N21A/F126A-ONDPA_pro*R*_, the conformations of ONDPA demonstrated a relatively close and stable distance to Tyr58 and NADPH. Remarkably, the statistical results showed that the proportion of catalytic conformation of *d* (O_sub_ − HO_Y58_) ≤ 3.4 Å and *d* (C_sub_ − H4_NADPH_) ≤ 4.5 Å in iolS^WT^-ONDPA_pro*S*_ was 2.5%, six times higher than that (0.4%) of iolS^WT^-ONDPA_pro*R*_ (Fig. 4A). Similarly, the catalytic conformation in I57F/F126L-ONDPA_pro*S*_ was 6.3%, approximately eight times that observed in I57F/F126L-ONDPA_pro*R*_ (0.8%) (Fig. 4B). The catalytic conformation of N21A/F126A-ONDPA_pro*R*_ was 9.2%, whereas the pro-*S* conformation was only 1.2%. Overall, the higher probability of prereaction configuration was conducive to the formation of the dominant conformation, which was consistent with the results of the experimental data (Fig. 4C). The above results demonstrated that I57F/F126L-ONDPA_pro*S*_ and N21A/F126A-ONDPA_pro*R*_ were favorable for the development of prereaction states to produce corresponding (*S*)-and (*R*)-NPHPA, respectively.

In order to analyze the role of key residues in the binding of ONDPA, per-residue free energy decomposition was performed using the MM-GBSA method in MD simulation of protein-ONDPA complexes [28]. For iolS^WT^, N21, I57, Y58 and F126 contributed more energy to binding of ONDPA_pro*S*_ than ONDPA_pro*R*_ (Fig. 4D). Except for the catalytic center Y58, the three amino acids were key residues that controlled the stereoselectivity of iolS. It indicated that residues offered more energy to binding of substrate could play crucial roles in the enantioselectivity of enzyme. In term of variant I57F/F126L, N21, F57 and L126 provided higher energy contributions for binding ONDPA_pro*S*_ (Fig. 4E). The introduction of F57 and L126 resulted in excellent stereoselectivity toward *S*-configuration. Notably, L126 provided the highest energy contribution to ONDPA_pro*S*_, which was a significant reason for the improved stereoselectivity of the variant. As shown in Fig. 4F, the residues A21, Y58, and A126 contributed more energy to binding of ONDPA_pro*R*_ than ONDPA_pro*S*_. Compare with N21A/F126A-ONDPA_pro*S*_, the energy contribution of N21A/F126A-ONDPA_pro*R*_ in the active site was more reasonable, which was beneficial to the stability of prereaction state and the occurrence of reduction. Interestingly, replacing N21 and F126 of iolS by A21 and A126 switched its selectivity toward ONDPA_pro*R*_. It revealed that sites 21 and 126 might work as a molecular switch in manipulating the stereopreference of iolS.

**Fig. 5:**
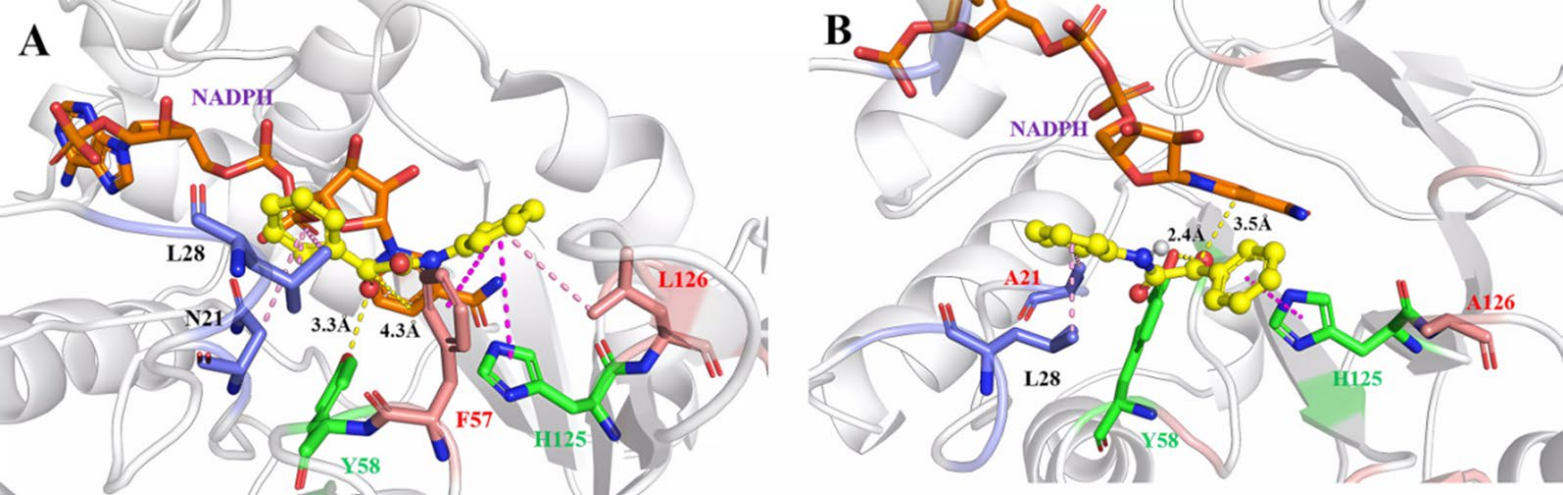
Binding modes of ONDPA to I57F/F126L and N21A/F126A in MD simulation. **A** Interactions between I57F/F126L and ONDPA_pro*S*_. **B** Interactions between N21A/F126A and ONDPA_pro*R*_

Furthermore, on the basis of docking the substrate ONDPA (1a) into iolS, variant I57F/F126L and N21A/F126A, the protein–ligand complexes were subjected to 50 ns MD simulation (Additional file 1: Fig. S3). After MD simulation, the average conformations of I57F/F126L-ONDPA_pro*S*_ and N21A/F126A-ONDPA_pro*R*_ in the prereaction state were extracted for further study (Fig. 5). As for the variant I57F/F126L, the hydrophobic interactions formed between ONDPA and I57F as well as F126L were considered to play a major role in substrate binding. The expansion of the large pocket space enhanced the interaction between ONDPA and the active site of iolS. *d* (O_sub_ − OH_Y58_) and *d* (C_sub_ − H4_NADPH_) were shortened to 3.3 Å and 4.3 Å, respectively, which facilitated hydrogen bond formation and hydride transfer. As for the variant N21A/F126A, the enlarged space of the small pocket could be favorable for the accommodation of the bulky substituent. The volume changes of N21 and F126 could influence the recognition of different orientations of substrates which was the main reason for the stereoselective inversion of N21A/F126A. In addition, the anti-Prelog conformations were also stabilized due to the disappearance of the original π − π interaction between site 126 and the *N*-phenylmethylamide group. Meanwhile, *d* (O_sub_ − HO_Y58_) and *d* (C_sub_ − H4_NADPH_) were shortened to within 2.4 Å and 3.5 Å, respectively, which was beneficial for the reduction of the carbonyl oxygen atom of ONDPA. This also successfully explained the high activity and stereoselectivity of variant N21A/F126A.

### Substrate spectrum analysis of iolS and variants

**Fig. 6:**
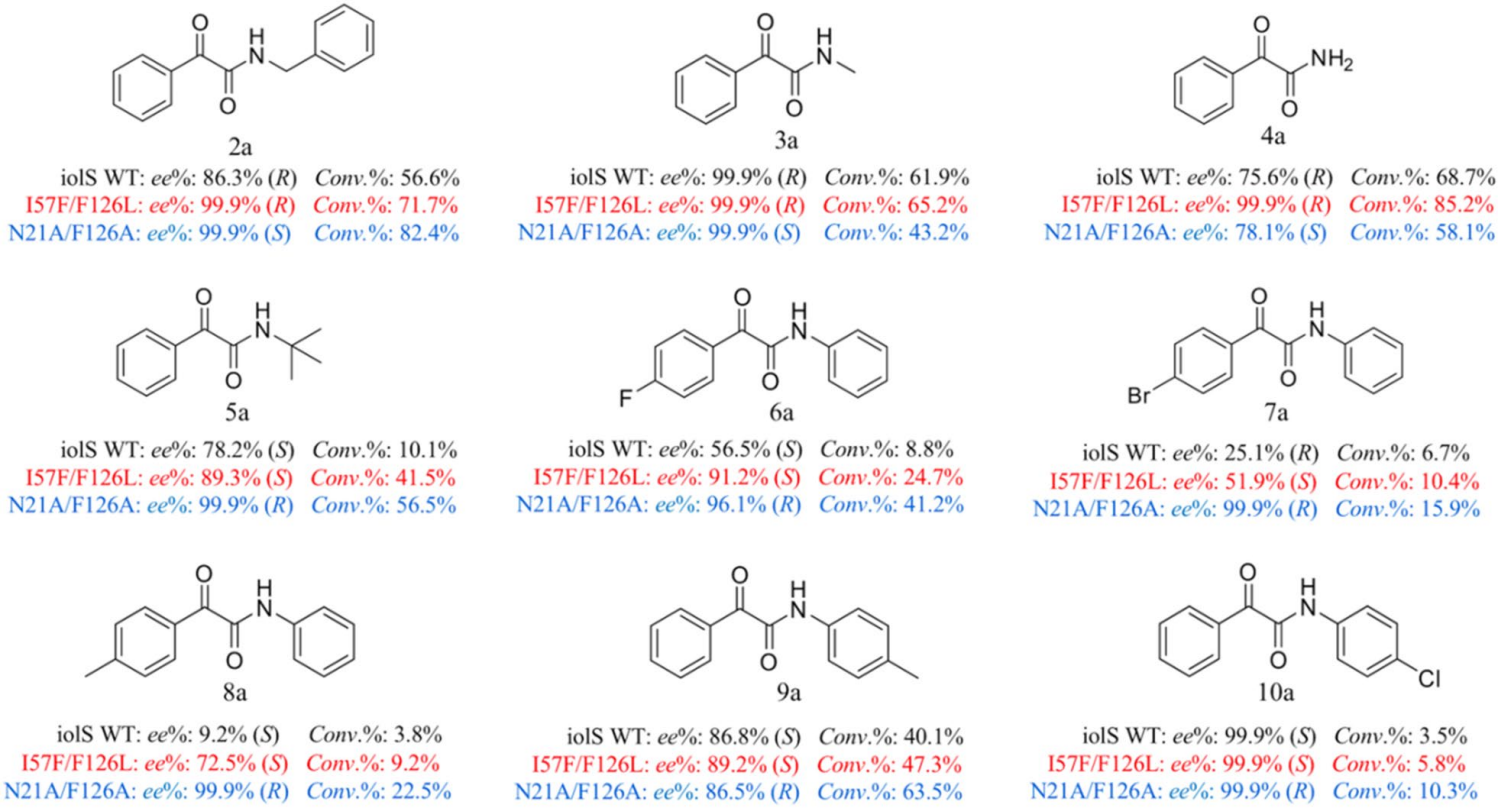
Substrate spectrum of iolS and variants toward various α-keto amides (**2a** − **10a**)

The application potential of iolS and its variants for the asymmetric synthesis of α-hydroxy amides was assessed using a variety of structural α-keto amides (**2a**–**10a**) (Fig. 6). For **2a**, the WT iolS exhibited a preference for the *R*-configuration, and the variant I57F/F126L (99.9%, *R*) could still improve its stereoselectivity, whereas N21A/F126A (99.9%, *S*) display reversed stereoselectivity. Substrates **3a**–**5a** (Fig. 6) belong to the *N*-alkyl α-keto amides. Compared with ONDPA (**1a**), the reversed configuration of **3a** and **4a** were due to the changes in group priority based on the Cahn − Ingold − Prelog rule. WT iolS exhibited a different conformation toward substrates with single bulky substituents, such as 99.9% (*R*) of **3a**, 75.6% (*R*) of **4a** and 78.1% (*S*) of **5a** in *ee* value. These results suggested that the volume of the substituents to which amide groups were attached was critical for stereoselective recognition of iolS. The asymmetric reduction toward **3a**–**5a** by Prelog variant I57F/F126L and anti-Prelog variant N21A/F126A gave the corresponding products with high *ee* values (up to > 99%). Moreover, in order to evaluate the function of the phenyl group in the recognition of ONDPA, the substrates with varied sizes and polarity substituents, such as *para*-fluoride-, *para*-bromide-, *para*-methyl-, and *para*-chloride-substituted substrates (**6a**–**10a**, Fig. 6) were investigated. The iolS displayed an opposite configuration in the reduction of **6a** (56.5%, *S*) and **7a** (25.1%, *R*). The stereoselectivity of WT with a poor *ee* value of 9.2% (*S*) toward 8a indicated that the phenyl group with methyl-substituted seems to have enormous implication on the enantioselectivity. The iolS-catalyzed asymmetric reduction of **9a** and **10a** also reveals that the electron-absorbing group located in *N-*phenylformamide group contributes to the improvement of the *ee* value of the reductive product, such as 86.8% (*S*) of **9a** and 99.9% (*S*) of **10a**. Evidently, electronic effects of substituents can affect the asymmetric reduction of diaryl α-keto amides (**6a**–**10a**) by iolS. The variants I57F/F126L and N21A/F126A exhibited outstanding enantioselectivity following the original *S-* or *R-*selectivity toward all *para*-substituent substrates as ONDPA. Finally, the stereoselectivities in asymmetric reduction by variant I57F/F126L toward **2a**, **3a**, **4a**, and **10a** were all above 99.9%. The variant N21A/F126A displayed 99.9% *ee* values toward **2a**, **3a**, **5a**, **7a**, **8a**, and **10a** (Fig. 6).

## Conclusions

In summary, this study proposed a novel enzyme-catalyzed route for the efficient synthesis of chiral α-hydroxy amides. The wild-type iolS exhibited a moderate *ee* (76.1%, *S*) and conversion (60.5%) in the reduction of ONDPA (**1a**). Two stereocomplementary variants, I57F/F126L and N21A/F126A were constructed by semi-rational engineering, which could asymmetrically synthesize NPHPA with *ee* values of 97.6% (*S*) and 99.9% (*R*), respectively, with both conversions reaching above 98%. Based on the molecular dynamics and docking analysis, the residues at 21 and 126 were identified as the "switch", which controlled the stereopreference of iolS between Prelog and anti-Prelog priorities by reshaping the substrate binding pocket. In MD simulation analysis, the variants I57F/F126L and N21A/F126A provided suitable binding pocket for accommodating the substrate with pro-*S* or pro-*R* catalytic configuration in the prereaction state, respectively. Substrate spectrum analysis revealed that two stereocomplementary variants I57F/F126L and N21A/F126A also exhibited high enantioselectivity toward the other α-keto amides. This study provides an effective strategy to produce optically pure α-hydroxy amides and offer the structural guidance for engineering enantiocomplementary AKRs toward α-keto amides.

## Methods

### Microorganism and chemistry

The primers of iolS were synthesized at Comate Bioscience Co. Ltd (Changchun, China). Restriction enzyme *Dpn*I and the primer STAR DNA polymerase were purchased from Takara Bio Inc. (Tokyo, Japan). High-performance liquid chromatography (HPLC)–grade hexane and 2-propanol were purchased from J&K Scientific Ltd. (Beijing, China). Recombinant *E. coli* Rosseta strains harboring pET28a-iolS and pET21a-GDH had been previously constructed in the laboratory [29].

### Synthesis of α-keto amides

SOCl_2_ (0.17 mL, 2.3 mmol) was added dropwise to the solution of phenylglyoxylic acid (0.296 g, 2 mmol) in *N*, *N*-dimethylaceamide (5 mL) cooled to -15 °C under a nitrogen atmosphere. The stirring for 1 h and then the aniline (0.25 mL, 2.78 mmol) was added slowly to the mixture. After stirring was continued at temperature between − 10 °C and 0 °C for 3 h, a mixture of ice and water was poured and stirred at room temperature overnight. The formation of product was monitored by TLC. The reaction mixture was extracted with ethyl acetate. The organic layer was washed with brine and water for three times, then dried over anhydrous Na_2_SO_4_ and concentrated in vacuo. The residue was purified by silica gel column chromatography [30]. All NMR data have been described in the Supporting Information (Additional file 1: Fig. S14–S23).

### Synthesis of racemic α-hydroxyl amines

Α-keto amides (4 mM) and NABH_4_ (4 mM) were added in 5 mL of tetrahydrofuran at 80 °C [31]. After stirring for 4 h, the reaction mixture was extracted with ethyl acetate. The organic layer was washed with brine and water for three times, then dried over anhydrous Na_2_SO_4_ and concentrated in vacuo. The residue was purified by silica gel column chromatography to give the corresponding racemic α-hydroxyl amines [10]. All NMR data have been described in the Supporting Information (Additional file 1: Fig. S14–S23).

### Site-directed/saturation mutagenesis

Site-directed/saturation mutagenesis of iolS was performed by whole plasmid PCR approach with PrimeSTAR DNA polymerase. The recombinant plasmid pET28a-iolS was used as the template for PCR to generate variations, and the associated primers have been presented in Additional file 1: Table S1. The PCR procedures were as follows: 98 °C for 3 min, followed by 30 cycles: 98 °C for 10 s, 60 °C for 15 s, and 72 °C for 6 min and 30 s, further elongation at 72 °C (10 min). The PCR products were validated by nucleic acid electrophoresis. The purified PCR product was digested with *Dpn*I. The digestive products were then immediately transformed into *E. coli* cells. Intended mutagenesis was confirmed by DNA sequencing at Comate Bioscience Co., Ltd. (Jilin, China).

### Expression and purification of iolS and variants

*E.coli* BL21(DE3) cells expressing iolS and variants genes were inoculated into LB medium containing 10 g/L of tryptone, 5 g/L of yeast extract, and 10 g/L of NaCl with 50 μg/mL kanamycin at 37 ℃ until the optical density was 0.6–0.8 at 600 nm. Enzyme expression was induced with isopropyl-*ß*-D-thiogalactopyranoside (IPTG, 0.1 mM) at 25 °C for 12 h. The cells were extracted by centrifugation at 15,285 × *g* for 5 min, resuspended in potassium phosphate buffer (100 mM, pH 7.0), and then subjected to ultrasonic cell disruption. Cell debris and intact cells were removed by centrifugation (15,285 × *g* for 10 min at 4 °C). Ni–NTA metal-affinity chromatography was used to purify the supernatants containing His-tagged iolS and variants.

### Kinetic assay

The kinetic parameters of iolS and variants were determined with the purified enzymes. The assay mixture (500 μL) contained 300 μL of pure enzymes (20 mg/mL), 10 μL of ONDPA (0.25–4.0 mM), 20 μL of NAD(P)H (4 mM), and 170 μL of potassium phosphate buffer (100 mM, pH 7.0). The reactions were conducted for 1 h at 30 °C and 190 rpm. The products were extracted with ethyl acetate for HPLC analysis. The kinetic parameters were calculated by measuring the initial velocities of the enzymatic reaction. The non-linear curve fitting was based on Michaelis − Menten equation using OriginPro (Version 8.5).

### Construction of the recombinant whole-cells

To construct the recombinant whole-cells containing iolS, or its variants and GDH, the double plasmids pET28a-iolS or -iolS variants and pET21a-GDH were co-transformed to BL21 (DE3) cells. In the LB medium contained 50 μg/mL kanamycin and 50 μg/mL ampicillin, the acquired iolS-GDH recombinant whole-cells were grown until the optical density reached 0.6 at 600 nm, and the final concentration of 0.1 mM IPTG was added. After the sample was incubated at 25 °C for 12 h, it was centrifuged at 15,285 × *g* for 4 min to obtain recombinant whole-cells.

### Whole-cell-catalyzed asymmetric reduction of α-keto amide compounds

Whole-cell-catalyzed asymmetric reduction of α-keto amides were carried out at 30 °C and 190 rpm. The reaction mixtures included recombinant whole-cells (wet weight 0.1 g), potassium phosphate buffer (100 mM, pH 7.0, 340 μL), α-keto amide compounds (**1a**–**10a**) (4.0 mM), glucose (200.0 mM), and DMSO (2%, v/v). The organic phase was extracted three times with ethyl acetate following a 3 h reaction. The conversion and *ee* value of α-hydroxy amides (1b–10b) were determined by HPLC with a Daicel Chiralpack OD-H or AD-H column (5 μm, 250 × 4.6 mm). Detailed conditions for specific methods and retention times have been presented in Additional file 1: Table S4. Chiral HPLC chromatograms are shown in Additional file 1: Fig. S4–S13.

### Molecular docking

The iolS crystal structure was taken from the Protein Data Bank (ID: 1PZ0) [32]. The enzyme molecule was optimized by using the force field CHARMm. The receptor proteins (iolS and variants) were prepared by removing all attached water molecules and adding hydrogen atoms [33]. Docking calculations were performed using AutoDock 1.5.6 (https://autodock.scripps.edu). The standard parameters were used for rigid receptor-flexible ligand docking. The iolS grid box dimensions and center were set as follows: center x, y, z = 4.439, 15.378, 69.361; size x, y, z = 40, 40, 40. The active pocket was defined within 4 Å of residues Asp53, Tyr58, Lys84, and His125 in the receptor protein molecule. The molecular docking poses were considered as reasonable according to hydrogen bond interactions between Tyr58-OH and carbonyl oxygen of ONDPA, as well as the distance of carbonyl carbon from the NADPH-C4 atoms being less than 4.5 Å.

### Molecular dynamic simulation of the prereaction state

Molecular dynamic (MD) simulation was performed using AMBER 18 (https://ambermd.org). The substrate and protein were described by a GAFF2 small molecule force field and ff14SB protein force field, respectively. Each system uses the LEaP module to add hydrogen atoms, truncated octahedral TIP3P solvent box 6 at 10 Å, and Na^+^/Cl^−^ in the system to balance the system charge, and finally output the topology and parameter files for simulation. The energy of the system was minimized before MD simulation. There were 2500 stages of steepest descent minimization and 2500 steps of conjugate gradient method in each minimization stage. After system energy optimization, the temperature of the system was slowly raised from 0 K to 298.15 K by heating the system for 200 ps at a constant volume and heating rate. Under the condition of NPT (isothermal isobaric), 500 ps equilibrium simulations were carried out for the whole system. Finally, the productive MD was performed for 50 ns in NPT ensemble at a temperature of 298.15 K. The energy decomposition of key residues in the substrate binding pocket to the binding of substrate using the MM/GBSA method in Amber18.

### Supplementary Information

**Additional file 1. **Additional tables and figures.

## Data Availability

The datasets supporting the conclusion of this article are included in the article and its additional files.

## Abbreviations

AKR: Aldo–keto reductases
ATH: Asymmetric transfer hydrogenation
GDH: Glucose dehydrogenase
HPLC: High-performance liquid chromatography
iolS: Type of AKR
IPTG: Isopropyl-ß-d-thio-galactopyranoside
MD: Molecular dynamic
NPHPA: *N*-Phenyl-2-hydroxy-2-phenylacetamide
NMR: Nuclear magnetic resonance
ONDPA: 2-Oxo*-N*, 2-diphenyl-acetamide
SDS-PAGE: Sodium dodecyl sulfate polyacrylamide gel electrophoresis
SM: Saturation mutagenesis
WT: Wild type
yhdN: Type of AKR
