# Supplementary material for: Semi-rational engineering an aldo–keto reductase for stereocomplementary reduction of α-keto amide compounds
Source: Microb Cell Fact. 2023 Oct 15;22:213. doi: 10.1186/s12934-023-02225-9 (PMC10577934; doi:10.1186/s12934-023-02225-9)
Supplement: Supplementary file 1 — Additional file 1. Additional tables and figures. [file 12934_2023_2225_MOESM1_ESM.docx]

Additional file 1

Semi-rational Engineering an Aldo-keto Reductase for Stereocomplementary Reduction of α-keto Amide Compounds

Ruixuan Bai^1^, Baoling Chen^2^, Liangyu Zheng*

Key Laboratory for Molecular Enzymology and Engineering of Ministry of Education, School of Life Sciences, Jilin University, Changchun 130012, China.

*Corresponding Author: Liangyu Zheng. Tel.: +86 431 85155252; fax: +86 431 85155252.

E-mail: zhengliangyu@jlu.edu.cn (L. Zheng).

**Table S1.** Primers for the site-saturation/directed mutagenesis of iolS.

| Strategy | Primer | Primer sequence (5’–3’) |
| --- | --- | --- |
| Alanine scanning | N21A-F | ATTAGGAACAGCTGCTGTCGGAGGACATAA |
|  | N21A-R | TTATGTCCTCCGACAGCAGCTGTTCCTAAT |
|  | N27A-F | GAGGACATGCTCTCTACCCGAACCTAAATG |
|  | N27A-R | CATTTAGGTTCGGGTAGAGAGCATGTCCTC |
|  | L28A-F | GAGGACATAACGCTTACCCGAACCTAAATG |
|  | L28A-R | CATTTAGGTTCGGGTAAGCGTTATGTCCTC |
|  | Y29A-F | GAGGACATAACCTCGCTCCGAACCTAAATG |
|  | Y29A-R | CATTTAGGTTCGGAGCGAGGTTATGTCCTC |
|  | I57A-F | ATGTTAGACACCGCTTACGCTTACGGGATC |
|  | I57A-R | GATCCCGTAAGCGTAAGCGGTGTCTAACAT |
|  | H87A-F | ATCGCGACAAAAGCCGCTGCTAGAAAACAA |
|  | H87A-R | TTGTTTTCTAGCAGCGGCTTTTGTCGCGAT |
|  | F94A-F | CAGAAAACAAGGCAATGACGCTGTCTTTGA |
|  | F94A-R | TCAAAGACAGCGTCATTGCCTTGTTTTCTG |
|  | F96A-F | CAGAAAACAAGGCAATGACTTTGTCGCTGA |
|  | F96A-R | TCAGCGACAAAGTCATTGCCTTGTTTTCTG |
|  | F126A-F | TTGTTCTACATTCACGCTCCTGACGAACAT |
|  | F126A-R | ATGTTCGTCAGGAGCGTGAATGTAGAACAA |
|  | N156A-F | TCCATCGGTGTATCCGCTTTCTCTTTAGAG |
|  | N156A-R | CTCTAAAGAGAAAGCGGATACACCGATGGA |
|  | Y203A-F | TATTTCATTTATCCCTGCTTTCCCTCTCGT |
|  | Y203A-R | ACGAGAGGGAAAGCAGGGATAAATGAAATA |
|  | L226A-F | TTCCCAGAAGGCGACGCTCGAAACGAACAG |
|  | L226A-R | CTGTTCGTTTCGAGCGTCGCCTTCTGGGAA |
| Saturation mutagenesis | N21X -F | ATTAGGAACANNKGCTGTCGGAGGACATAA |
|  | N21X -R | TTATGTCCTCCGACAGCMNNTGTTCCTAAT |
|  | L28X-F | GAGGACATAACNNKTACCCGAACCTAAATG |
|  | L28X-R | CATTTAGGTTCGGGTAMNNGTTATGTCCTC |
|  | Y29X-F | GAGGACATAACCTCNNKCCGAACCTAAATG |
|  | Y29X-R | CATTTAGGTTCGGMNNGAGGTTATGTCCTC |
|  | I57X-F | ATGTTAGACACCGCTTACNNKTACGGGATC |
|  | I57X-R | GATCCCGTAMNNGTAAGCGGTGTCTAACAT |
|  | H87X-F | ATCGCGACAAAAGCCGCTNNKAGAAAACAA |
|  | H87X-R | TTGTTTTCTMNNAGCGGCTTTTGTCGCGAT |
|  | F126X-F | TTGTTCTACATTCACNNKCCTGACGAACAT |
|  | F126X-R | ATGTTCGTCAGGMNNGTGAATGTAGAACAA |

**Table S2** Asymmetric reduction of ONDPA catalyzed by free AKRs and AKRs-GDH whole cells.

| Form | T (h) | iolS | | yhdN | |
| --- | --- | --- | --- | --- | --- |
|  |  | *ee* (%) | *Conv.* (%) | *ee* (%) | *Conv.* (%) |
| Purified enzyme | 12 | 76.0 ± 0.4 (*S*) | 40.8 ± 0.5 | 3.3 ± 0.1 (*S*) | 35.1 ± 0.3 |
| whole-cell | 3 | 76.1 ± 0.6 (*S*) | 60.5 ± 0.5 | 4.5 ± 0.2 (*S*) | 48.4 ± 0.6 |

**Table S3** Saturation mutagenesis of single amino acid residue using NNK codon degeneracy with wild iolS as a template.

| Variants | *ee* (%) | *Conv.* (%) | Favored enantiomer | Variants | *ee* (%) | *Conv.* (%) | Favored enantiomer |
| --- | --- | --- | --- | --- | --- | --- | --- |
| N21K | 17.9 ± 0.2 | 88.1 ± 0.8 | *R* | I57T | 38.0 ± 1.1 | 85.9 ± 1.2 | *S* |
| N21Q | 7.7 ± 0.8 | 48.3 ± 1.3 | *R* | I57S | 11.5 ± 0.6 | 57.2 ± 0.6 | *S* |
| N21V | 34.4 ± 0.5 | 76.8 ± 0.6 | *R* | I57D | 8.8 ± 0.4 | 81.7 ± 1.0 | *R* |
| N21T | 28.1 ± 0.5 | 76.3 ± 0.8 | *R* | I57G | 1.1 ± 0.5 | 43.8 ± 1.3 | *R* |
| N21L | 7.7 ± 0.3 | 48.2 ± 1.2 | *R* | I57F | 90.6 ± 0.5 | 93.2 ± 1.4 | *S* |
| N21S | 88.4 ± 0.3 | 93.4 ± 0.4 | *R* | I57Y | 89.9 ± 0.4 | 83.0 ± 1.3 | *S* |
| N21D | 0.7 ± 0.2 | 40.2 ± 1.0 | *R* | I57H | 87.3 ± 0.8 | 76.9 ± 1.6 | *S* |
| N21I | 31.6 ± 0.8 | 61.7 ± 1.3 | *R* | H87V | 14.8 ± 0.7 | 55.8 ± 1.4 | *R* |
| N21G | 3.4 ± 0.2 | 64.7 ± 1.0 | *R* | H87L | 5.5 ± 0.4 | 49.7 ± 1.1 | *R* |
| L28G | 18.2 ± 0.7 | 58.2 ± 0.9 | *R* | H87F | 0.5 ± 0.5 | 77.0 ± 0.7 | *S* |
| L28S | 16.3 ± 0.5 | 52.0 ± 1.0 | *R* | H87Y | 1.1 ± 0.2 | 43.8 ± 1.5 | *R* |
| L28I | 62.6 ± 0.4 | 62.0 ± 1.3 | *S* | F126G | 8.4 ± 0.3 | 56.7 ± 1.1 | *R* |
| L28V | 61.1 ± 0.6 | 72.2 ± 1.2 | *S* | F126S | 13.9 ± 0.6 | 50.3 ± 1.4 | *R* |
| L28F | 63.1 ± 0.8 | 61.4 ± 1.0 | *S* | F126V | 86.2 ± 0.8 | 91.4 ± 1.5 | *S* |
| L28N | 37.2 ± 0.6 | 50.6 ± 1.2 | *S* | F126N | 74.2 ± 0.7 | 73.1 ± 0.9 | *S* |
| Y29F | 26.4 ± 0.8 | 74.3 ± 1.0 | *S* | F126H | 57.4 ± 0.3 | 58.2 ± 1.1 | *S* |
| Y29S | 15.0 ± 0.4 | 68.2 ± 1.3 | *R* | F126W | 54.2 ± 0.4 | 61.2 ± 1.4 | *S* |
| Y29V | 12.1 ± 0.2 | 53.1 ± 1.1 | *R* | F126Y | 57.3 ± 0.6 | 75.3 ± 1.5 | *S* |
| Y29H | 57.6 ± 0.2 | 71.2 ± 1.4 | *S* | F126T | 91.5 ± 0.8 | 91.3 ± 1.4 | *S* |
| Y29R | 27.6 ± 0.2 | 47.3 ± 1.3 | *S* | F126L | 95.3 ± 1.2 | 95.0 ± 1.1 | *S* |
| Y29G | 17.7 ± 0.3 | 62.4 ± 1.1 | *R* | F126I | 94.0 ± 1.0 | 92.8 ± 1.5 | *S* |

**Table S4.** HPLC conditions and retention times for enantiomers of α-hydroxy amide 1b-10b.

| Hydroxy amides | Column | Mobile phase | Retention time |
| --- | --- | --- | --- |
| (*R, S*)-1b | Chiralcel OD-H column (0.46 mm×250 mm, 5 µm) | n-hexane: isopropanol=90:10 | (*R*)-1b: 14.6 min, (*S*)-1b: 28.6 min |
| (*R, S*)-2b | Chiralcel OD-H column (0.46 mm×250 mm, 5 µm) | n-hexane: isopropanol=95:5 | (*R*)-2b: 33.7 min, (*S*)-2b: 36.6 min |
| (*R, S*)-3b | Chiralcel OD-H column (0.46 mm×250 mm, 5 µm) | n-hexane: isopropanol=95:5 | (*R*)-3b: 25.1 min, (*S*)-3b: 20.6 min |
| (*R, S*)-4b | Chiralcel OD-H column (0.46 mm×250 mm, 5 µm) | n-hexane: isopropanol=95:5 | (*R*)-4b: 46.5 min, (*S*)-4b: 42.2 min |
| (*R, S*)-5b | Chiralcel AD-H column (0.46 mm×250 mm, 5 µm) | n-hexane: isopropanol=95:5 | (*R*)-5b: 10.6 min, (*S*)-5b: 9.0 min |
| (*R, S*)-6b | Chiralcel OD-H column (0.46 mm×250 mm, 5 µm) | n-hexane: isopropanol=90:10 | (*R*)-6b: 15.5 min, (*S*)-1b: 33.7 min |
| (*R, S*)-7b | Chiralcel OD-H column (0.46 mm×250 mm, 5 µm) | n-hexane: isopropanol=90:10 | (*R*)-7b: 15.0 min, (*S*)-7b: 29.0 min |
| (*R, S*)-8b | Chiralcel OD-H column (0.46 mm×250 mm, 5 µm) | n-hexane: isopropanol=90:10 | (*R*)-8b: 13.8 min, (*S*)-8b: 25.3 min |
| (*R, S*)-9b | Chiralcel OD-H column (0.46 mm×250 mm, 5 µm) | n-hexane: isopropanol=90:10 | (*R*)-9b: 16.0 min, (*S*)-9b: 19.4 min |
| (*R, S*)-10b | Chiralcel OD-H column (0.46 mm×250 mm, 5 µm) | n-hexane: isopropanol=90:10 | (*R*)-10b: 11.2 min, (*S*)-10b: 12.1 min |

**
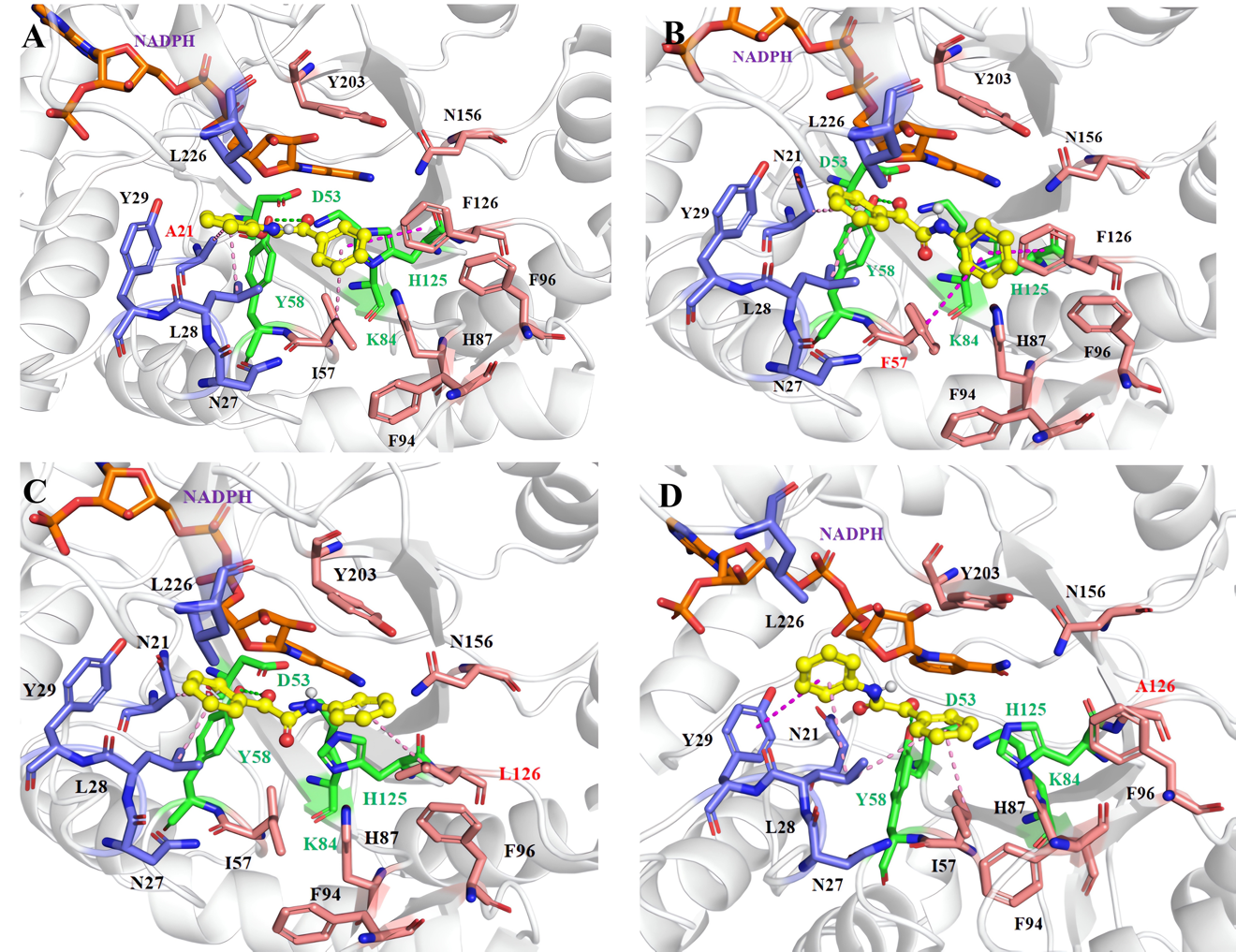
**

**Figure S1.** Docking poses of ONDPA in variant N21A (A), variant I57F (B), variant F126L (C) and variant F126A (D). Yellow: ONDPA; orange: NADPH; residues of binding pockets are shown by sticks; green: hydrogen bonds; magenta: π–π interactions; pink: π–σ and π–Alkyl interactions.

**
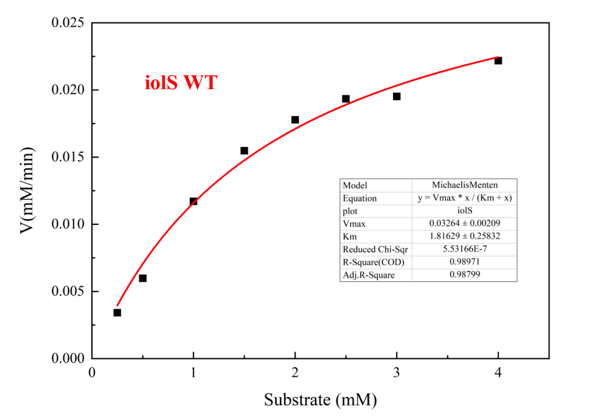

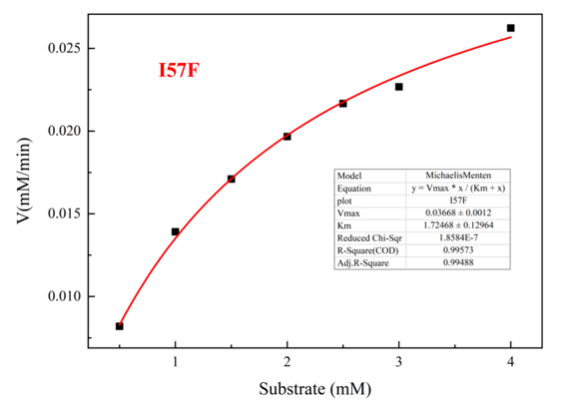
**

**
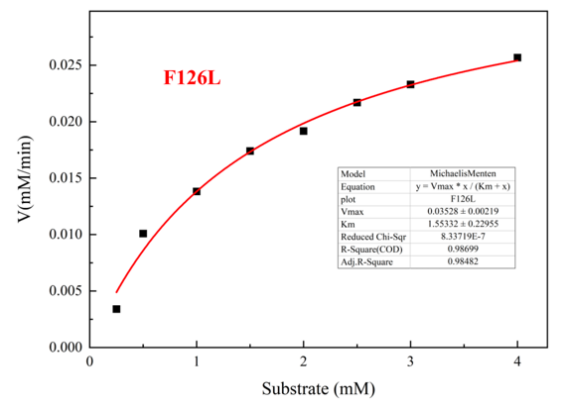
**
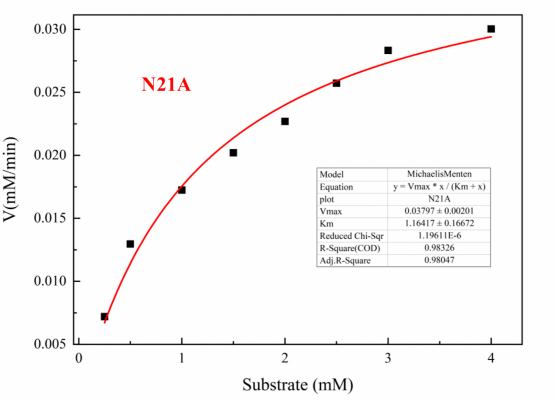


**
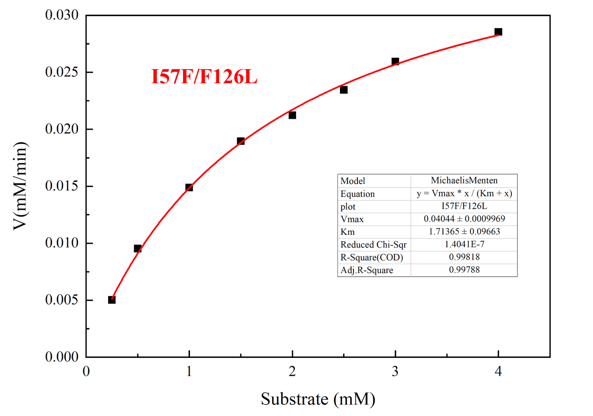

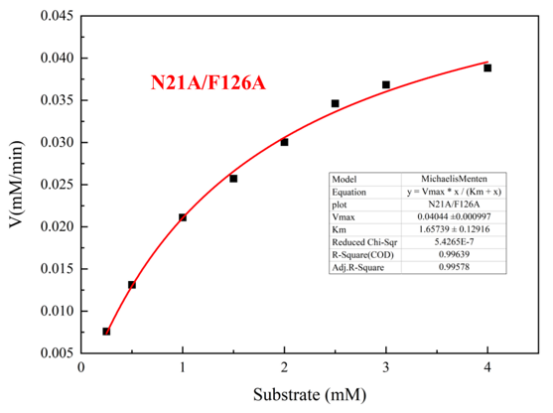
**

**Figure S2.** Kinetic parameters of iolS- or its variant-catalyzed asymmetric reduction reaction of ONDPA.

**Figure S3.** Total energy and RMSD of protein-ONDPA complexes in 50 ns MD simulation.

**
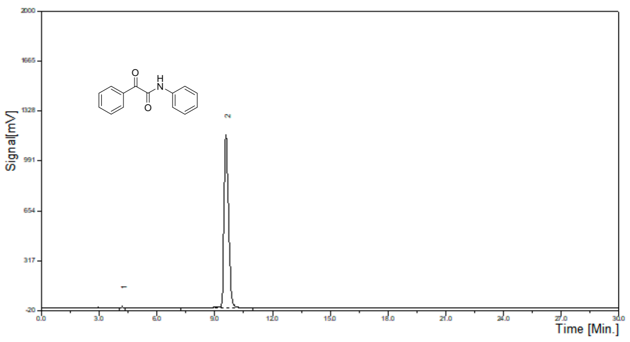

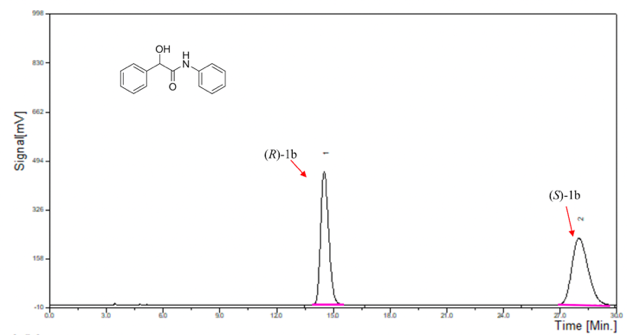
**

**
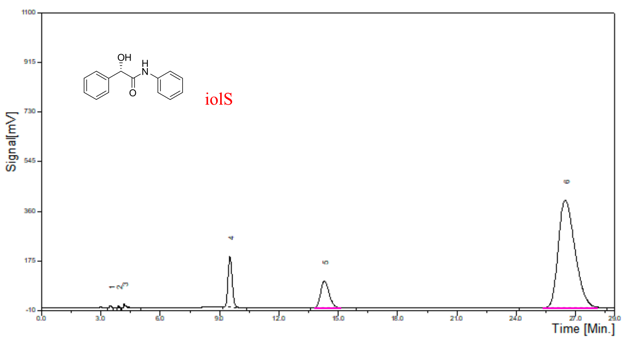

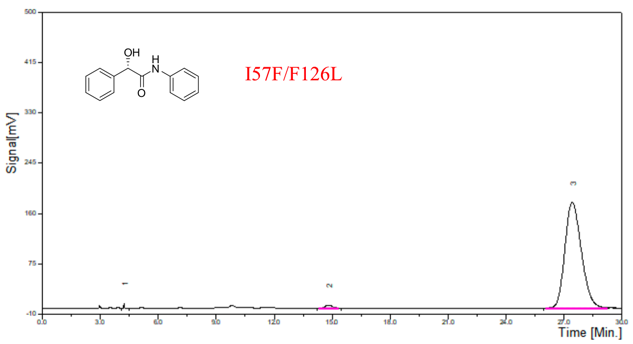
**


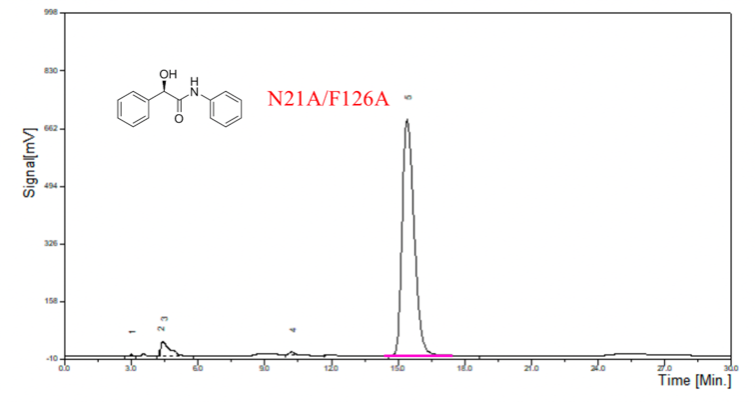


**Figure S4.** HPLC spectra of **1a**, **1b** (racemic), iolS-, I57F/F126L- and N21A/F126A-catalyzed asymmetric reduction reaction toward **1a**, respectively.

**
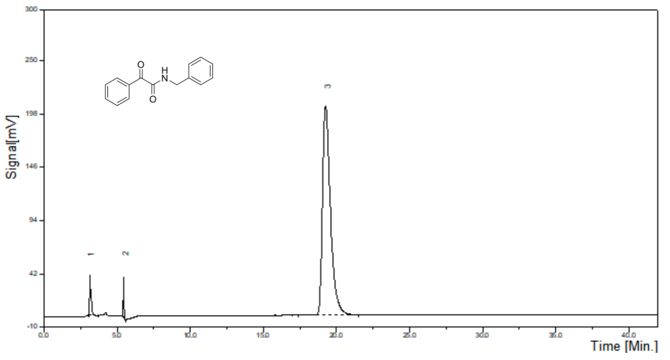

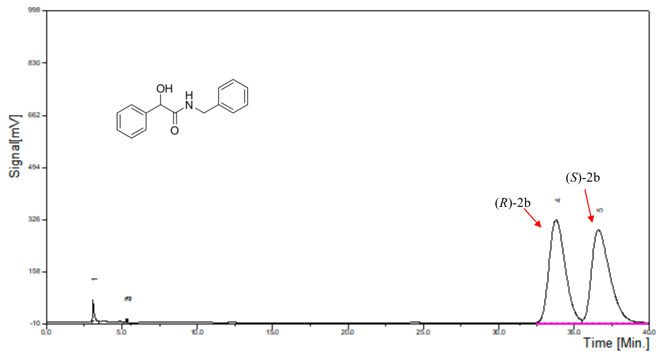
**

**
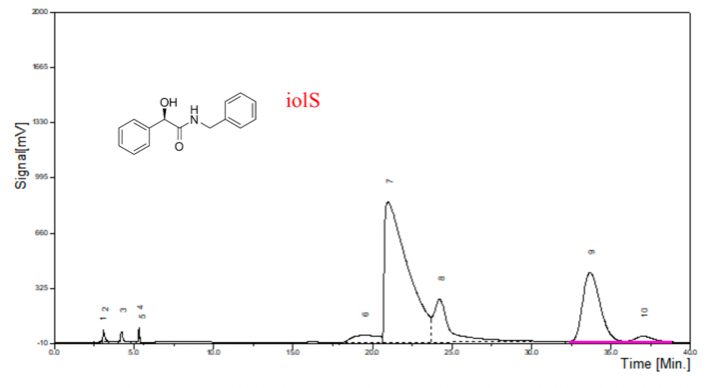

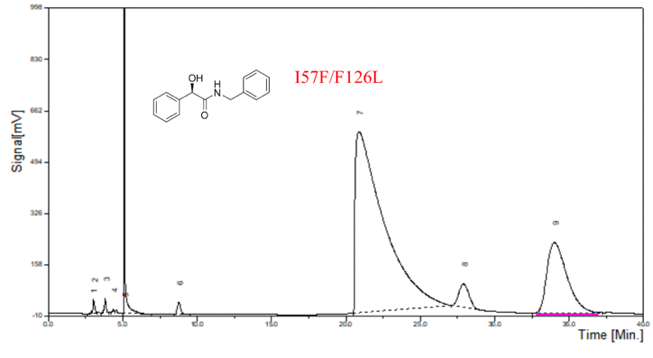
**

**
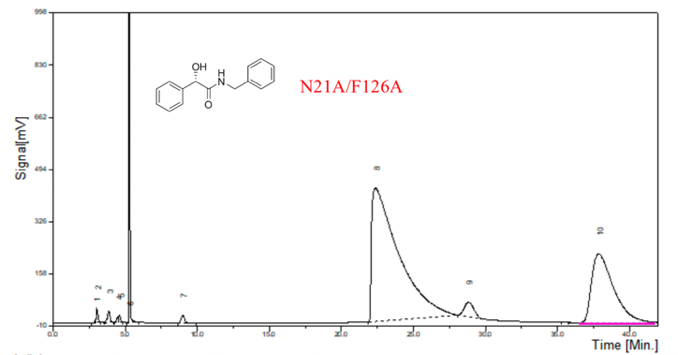
**

**Figure S5.** HPLC spectra of **2a**, **2b** (racemic), iolS-, I57F/F126L- and N21A/F126A-catalyzed asymmetric reduction reaction toward **2a**, respectively.


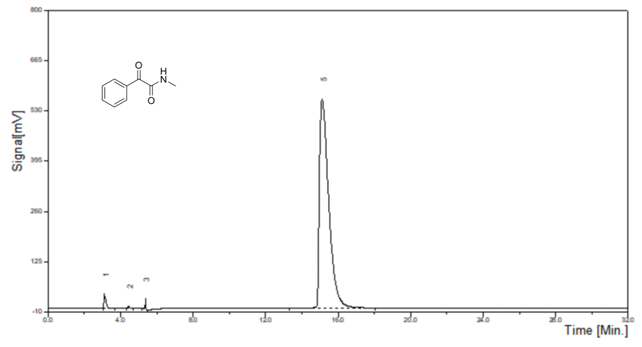

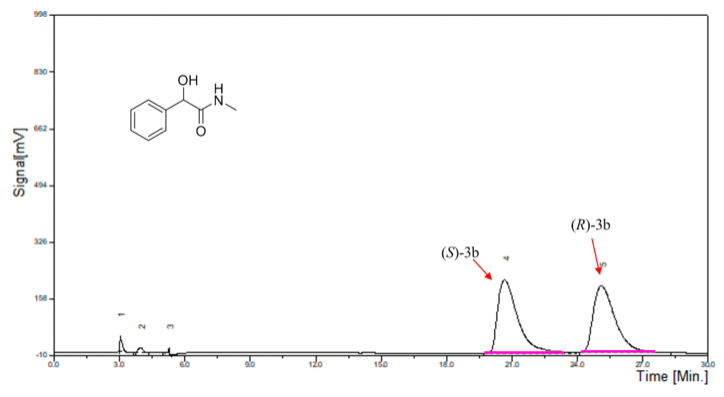

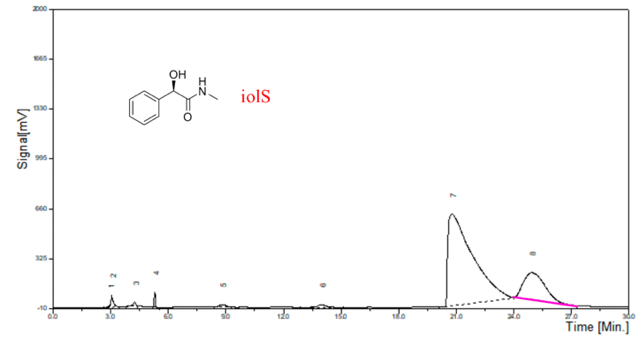

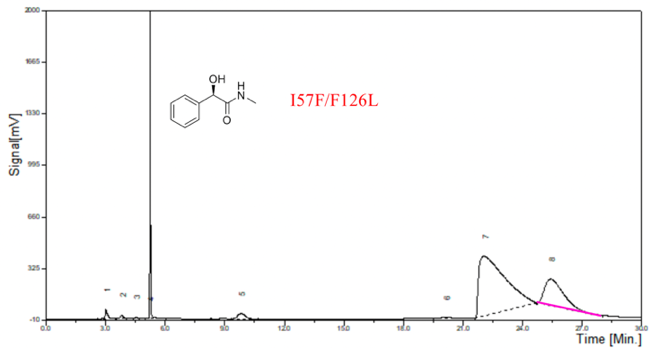


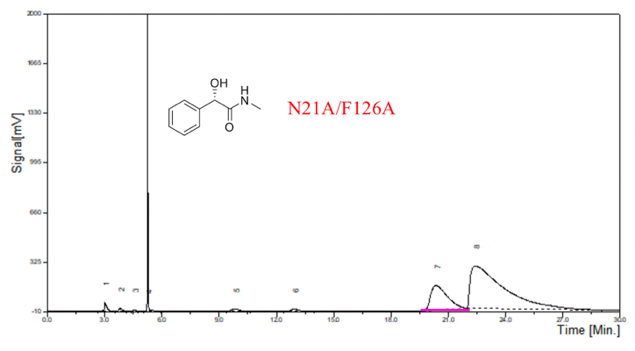


**Figure S6.** HPLC spectra of **3a**, **3b** (racemic), iolS-, I57F/F126L- and N21A/F126A-catalyzed asymmetric reduction reaction toward **3a**, respectively.


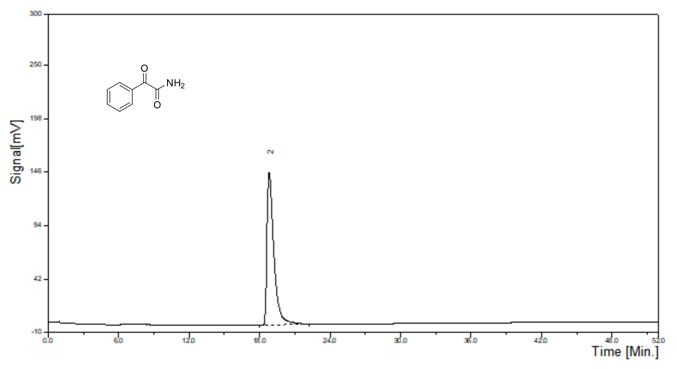

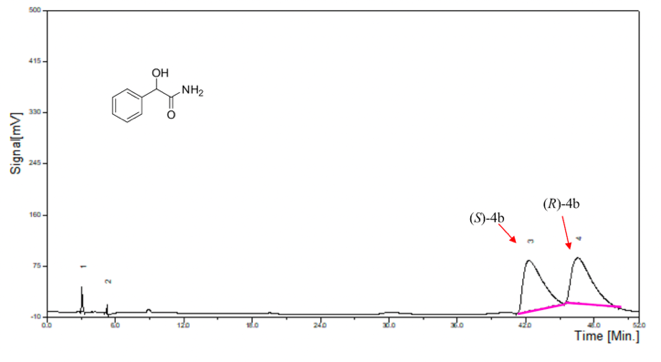

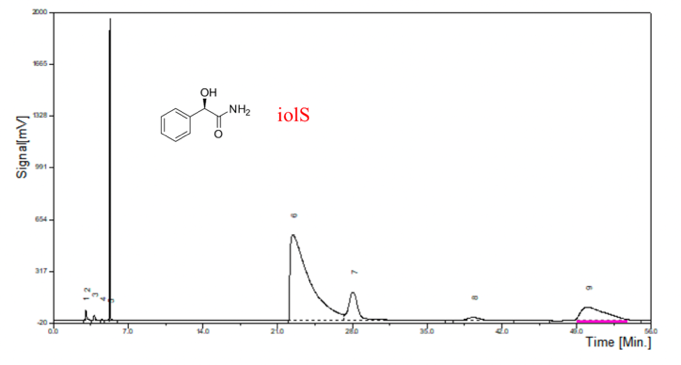

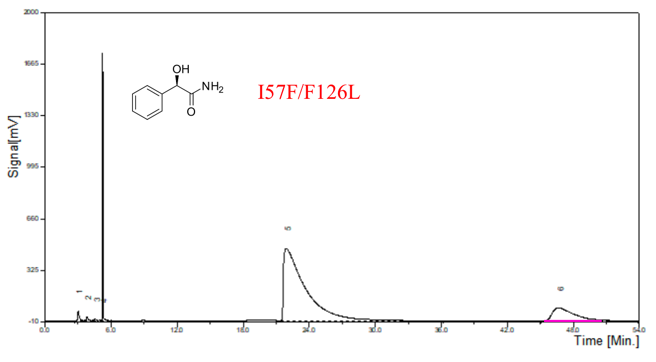


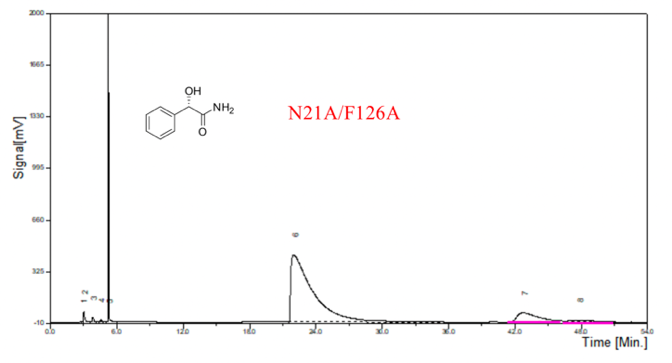


**Figure S7.** HPLC spectra of **4a**, **4b** (racemic), iolS-, I57F/F126L- and N21A/F126A-catalyzed asymmetric reduction reaction toward **4a**, respectively.


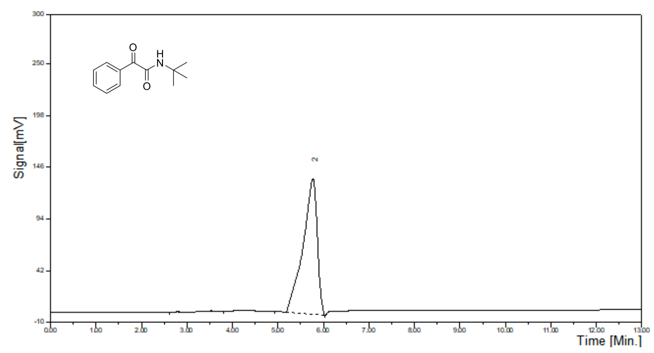

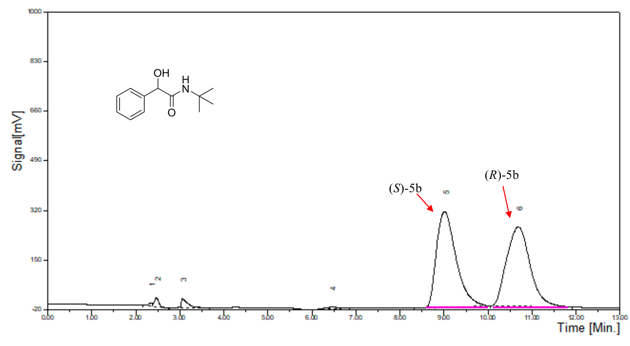

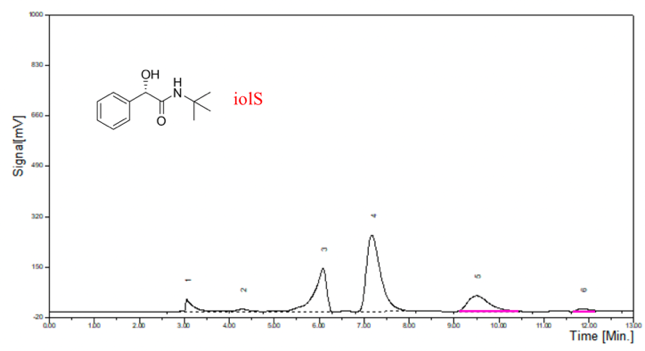

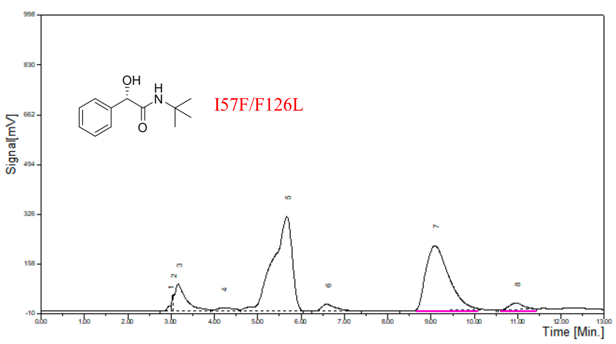


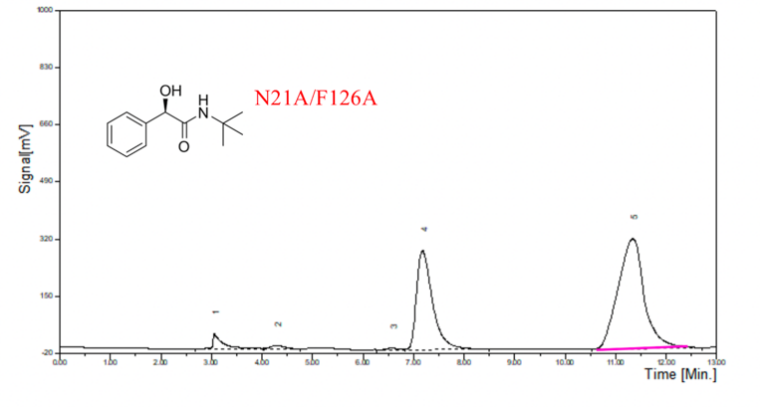


**Figure S8.** HPLC spectra of **5a**, **5b** (racemic), iolS-, I57F/F126L- and N21A/F126A-catalyzed asymmetric reduction reaction toward **5a**, respectively.


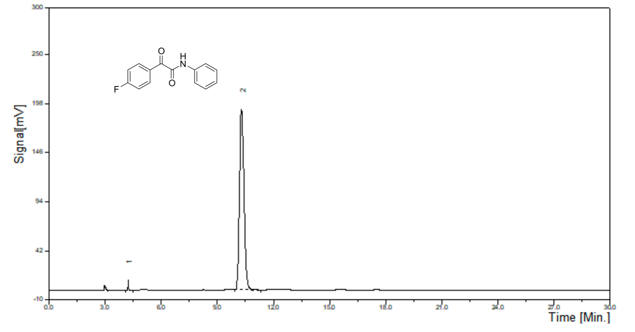

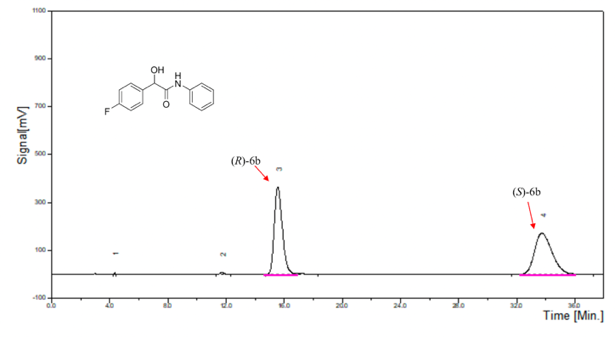

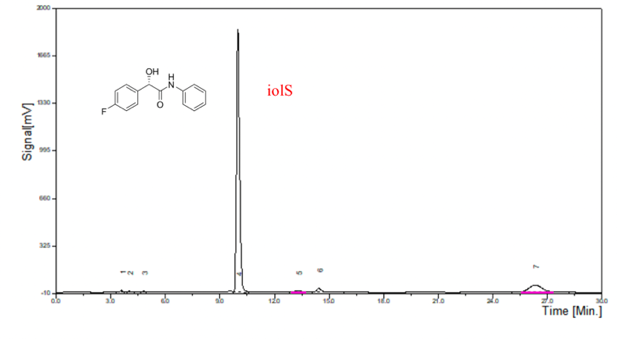

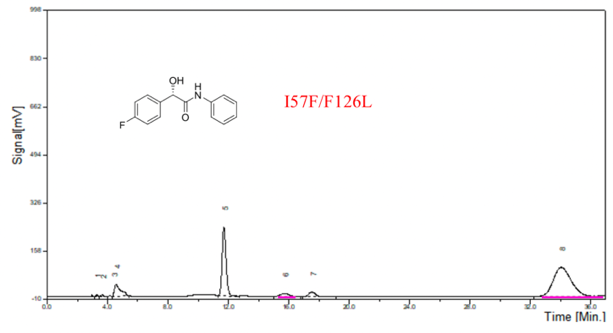


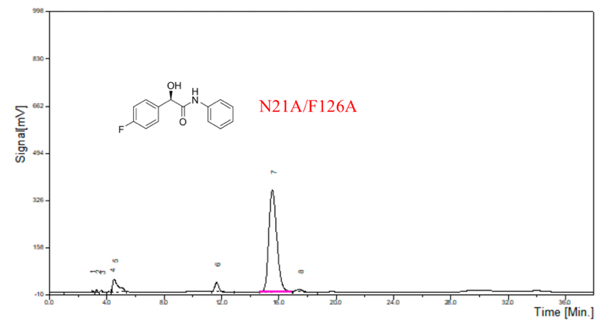


**Figure S9.** HPLC spectra of **6a**, **6b** (racemic), iolS-, I57F/F126L- and N21A/F126A-catalyzed asymmetric reduction reaction toward **6a**, respectively.


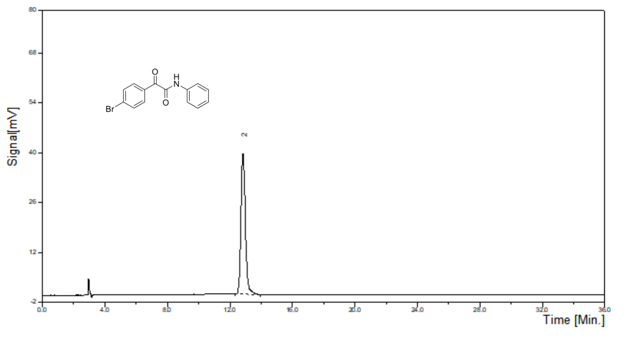

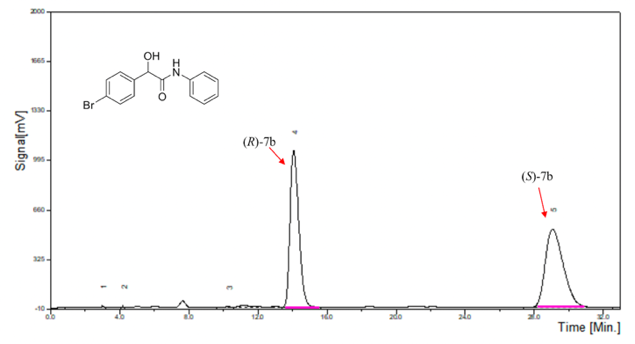


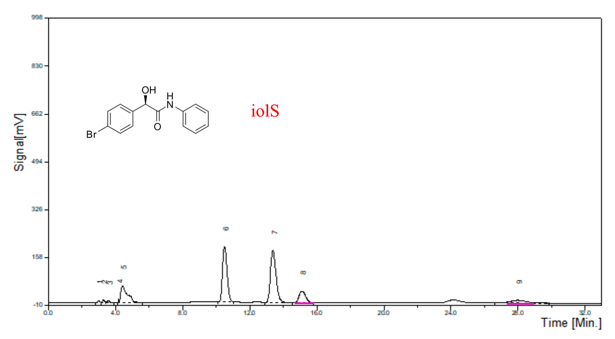

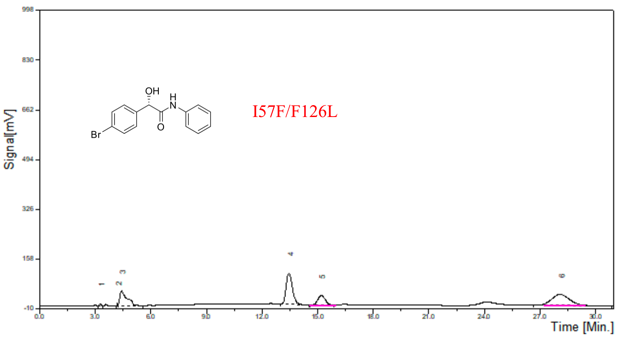


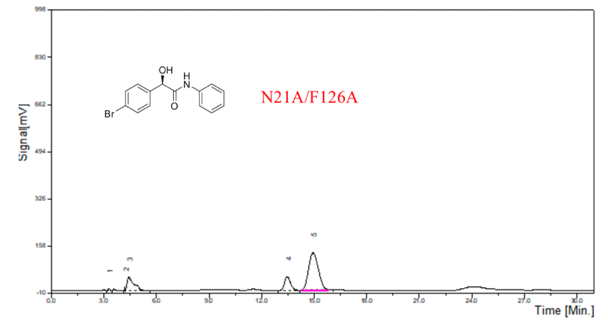


**Figure S10.** HPLC spectra of **7a**, **7b** (racemic), iolS-, I57F/F126L- and N21A/F126A-catalyzed asymmetric reduction reaction toward **7a**, respectively.


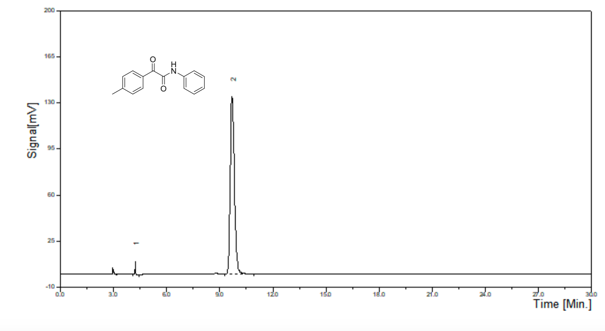

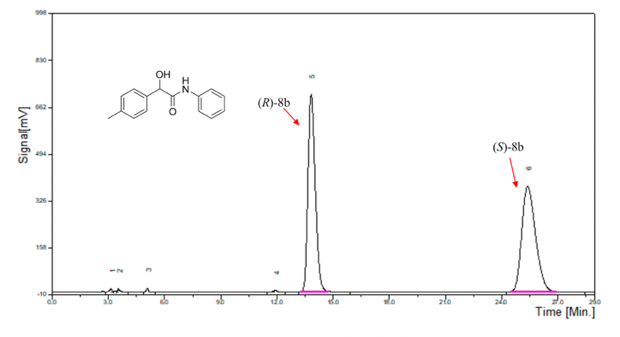


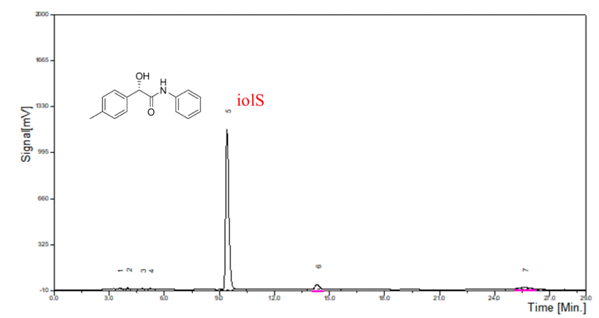

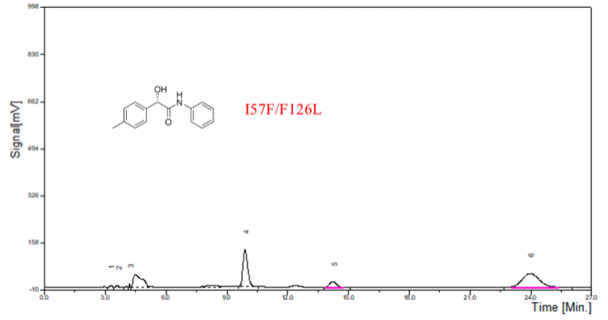


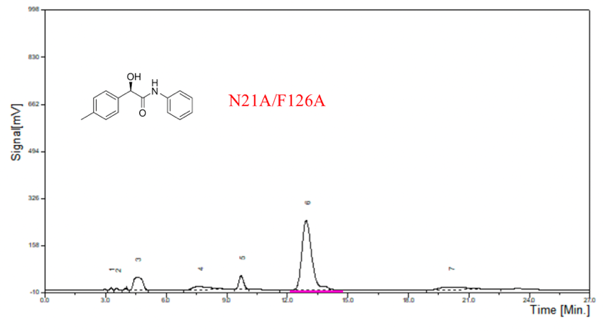


**Figure S11.** HPLC spectra of **8a**, **8b** (racemic), iolS-, I57F/F126L- and N21A/F126A-catalyzed asymmetric reduction reaction toward **8a**, respectively.


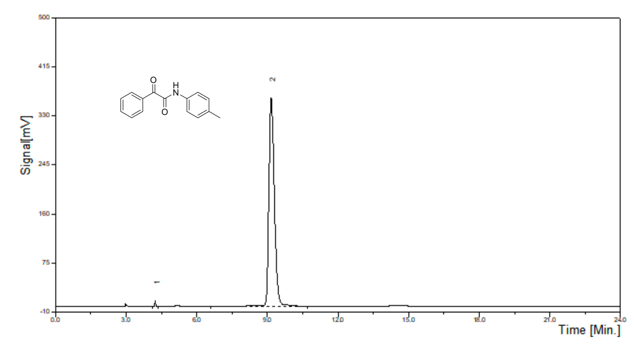

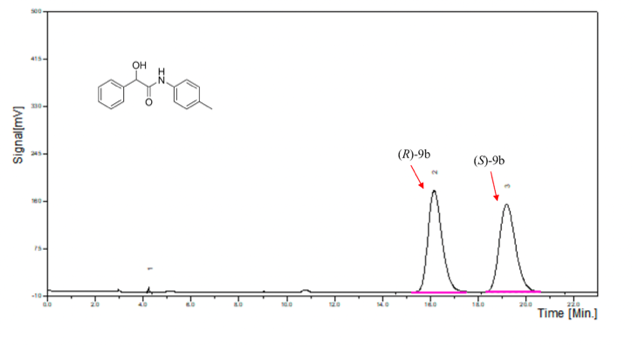

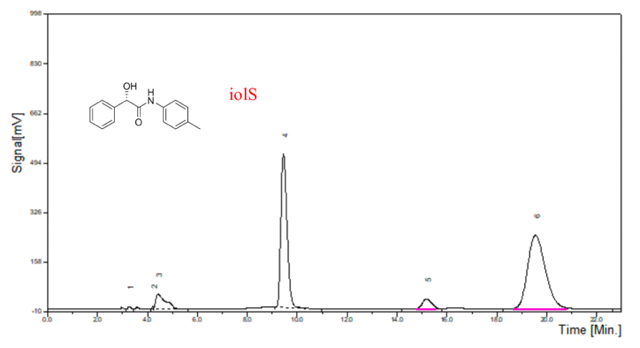

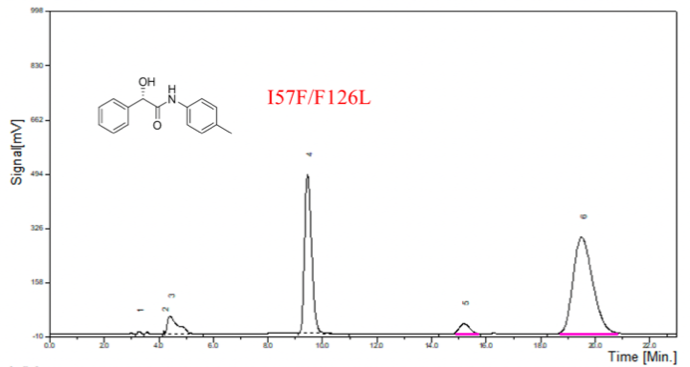

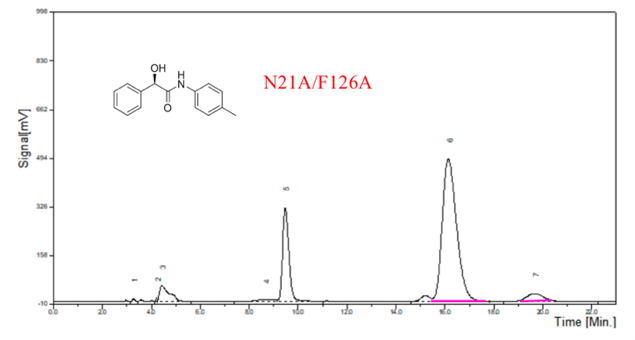


**Figure S12.** HPLC spectra of **9a**, **9b** (racemic), iolS-, I57F/F126L- and N21A/F126A-catalyzed asymmetric reduction reaction toward **9a**, respectively.


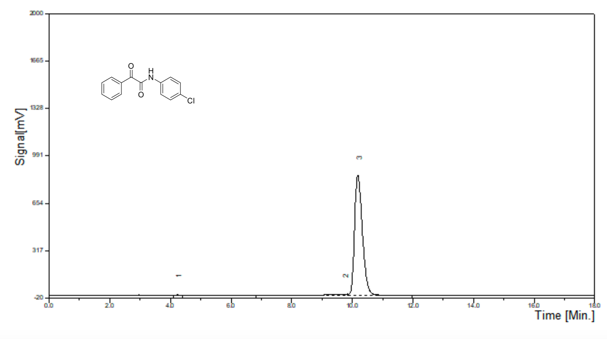

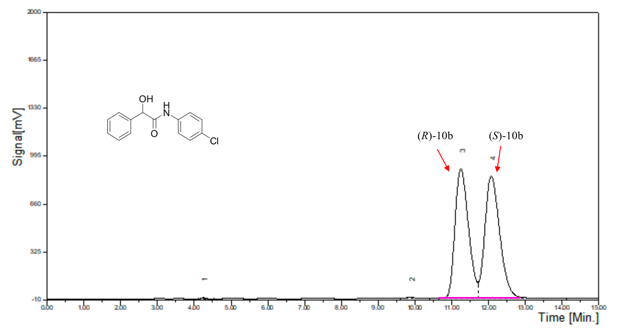


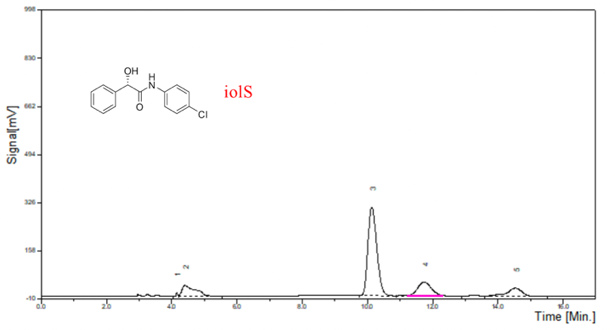

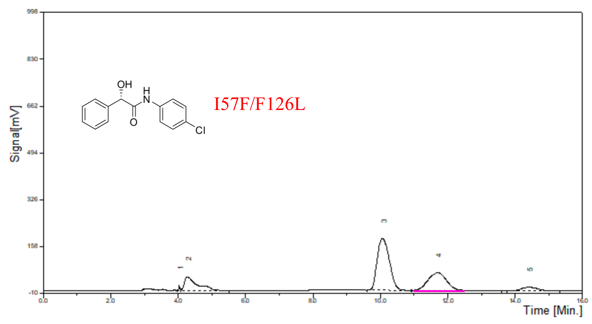


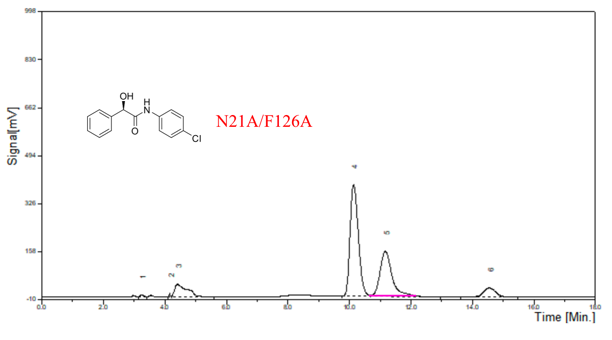


**Figure S13.** HPLC spectra of **10a**, **10b** (racemic), iolS-, I57F/F126L- and N21A/F126A-catalyzed asymmetric reduction reaction toward **10a**, respectively.


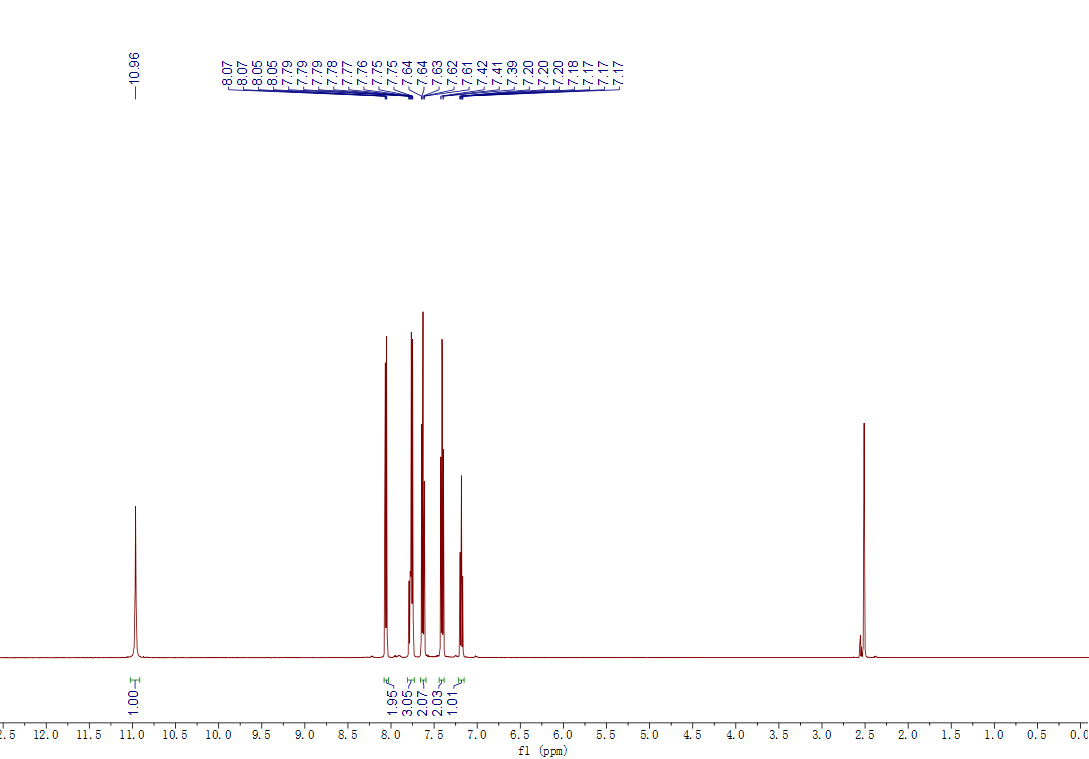


**1a**


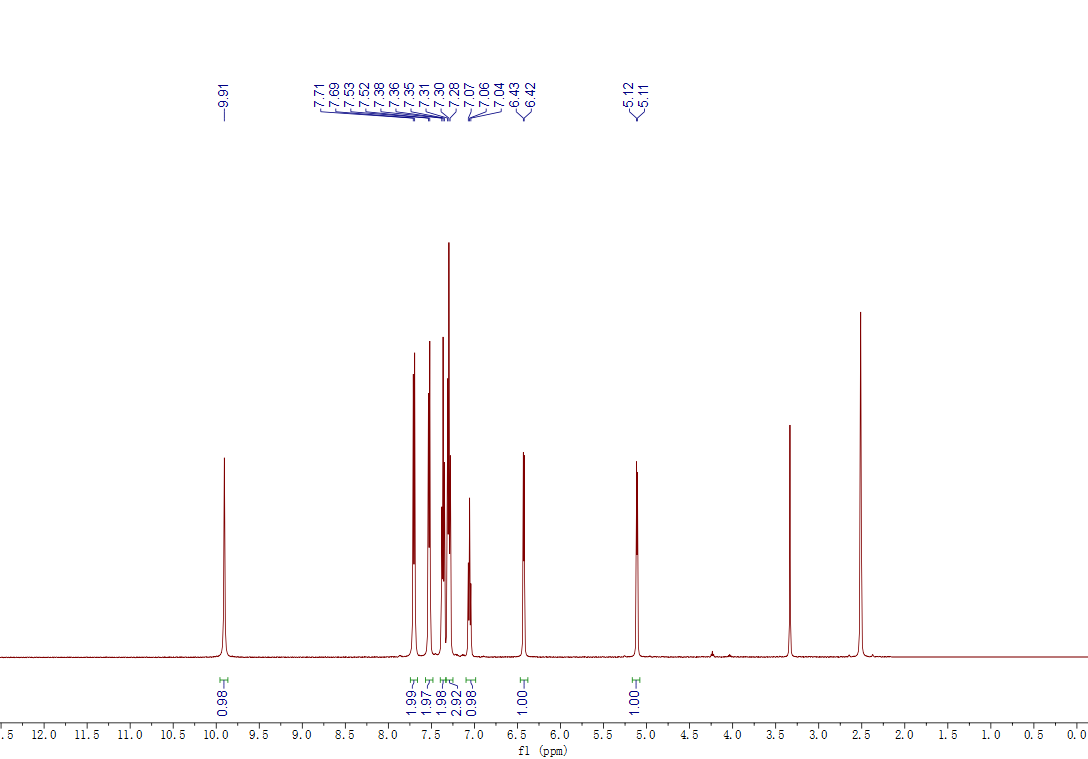


**1b**

**Figure S14**. The ^1^H NMR spectrum of **1a** and **1b**. **1a** ^1^H NMR (500 MHz, DMSO-*d*_6_) δ 10.96 (s, 1H), 8.06 (dd, *J* = 8.3, 1.4 Hz, 2H), 7.81 – 7.73 (m, 3H), 7.63 (t, *J* = 7.8 Hz, 2H), 7.44 – 7.38 (m, 2H), 7.22 – 7.15 (m, 1H). **1b** ^1^H NMR (500 MHz, DMSO-*d*_6_) δ 9.91 (s, 1H), 7.70 (d, *J* = 8.0 Hz, 2H), 7.53 (d, *J* = 7.5 Hz, 2H), 7.36 (t, *J* = 7.5 Hz, 2H), 7.30 (d, *J* = 7.6 Hz, 3H), 7.06 (t, *J* = 7.4 Hz, 1H), 6.43 (d, *J* = 4.7 Hz, 1H), 5.11 (d, *J* = 4.7 Hz, 1H).


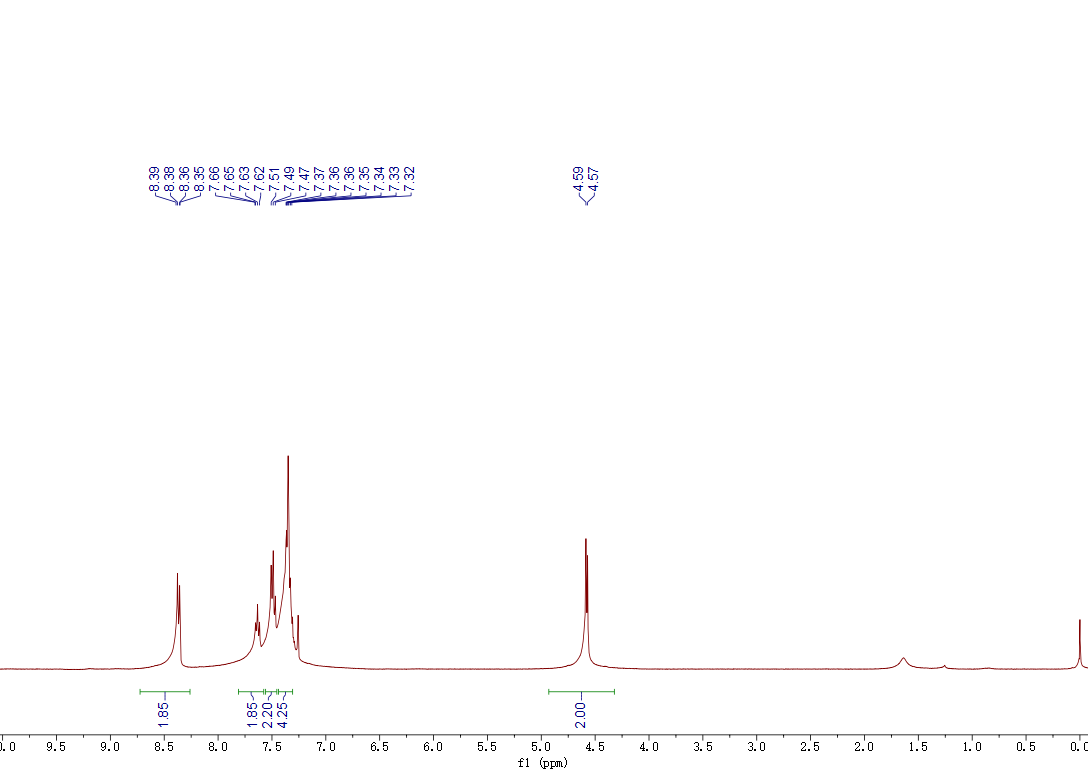


**2a**


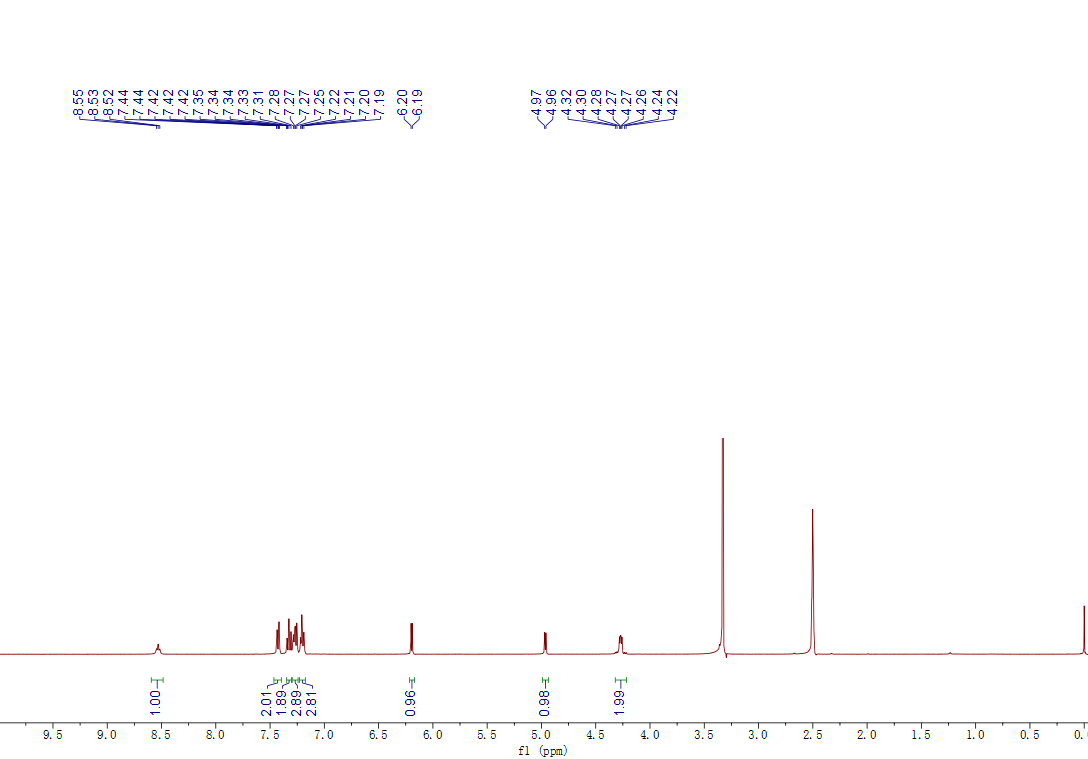


**2b**

**Figure S15**. The ^1^H NMR spectrum of **2a** and **2b**. **2a** ^1^H NMR (400 MHz, Chloroform-*d*) δ 8.72 – 8.26 (m, 2H), 7.81 – 7.58 (m, 2H), 7.49 (t, *J* = 7.6 Hz, 2H), 7.44 – 7.29 (m, 4H), 4.58 (d, *J* = 6.1 Hz, 2H).

**2b** ^1^H NMR (400 MHz, DMSO-*d*_6_) δ 8.53 (t, *J* = 6.3 Hz, 1H), 7.46 – 7.39 (m, 2H), 7.33 (t, *J* = 7.3 Hz, 3H), 7.27 (dd, *J* = 7.1, 5.2 Hz, 4H), 7.20 (dd, *J* = 7.1, 4.9 Hz, 3H), 6.20 (d, *J* = 4.7 Hz, 1H), 4.96 (d, *J* = 4.7 Hz, 1H), 4.32 – 4.22 (m, 2H).


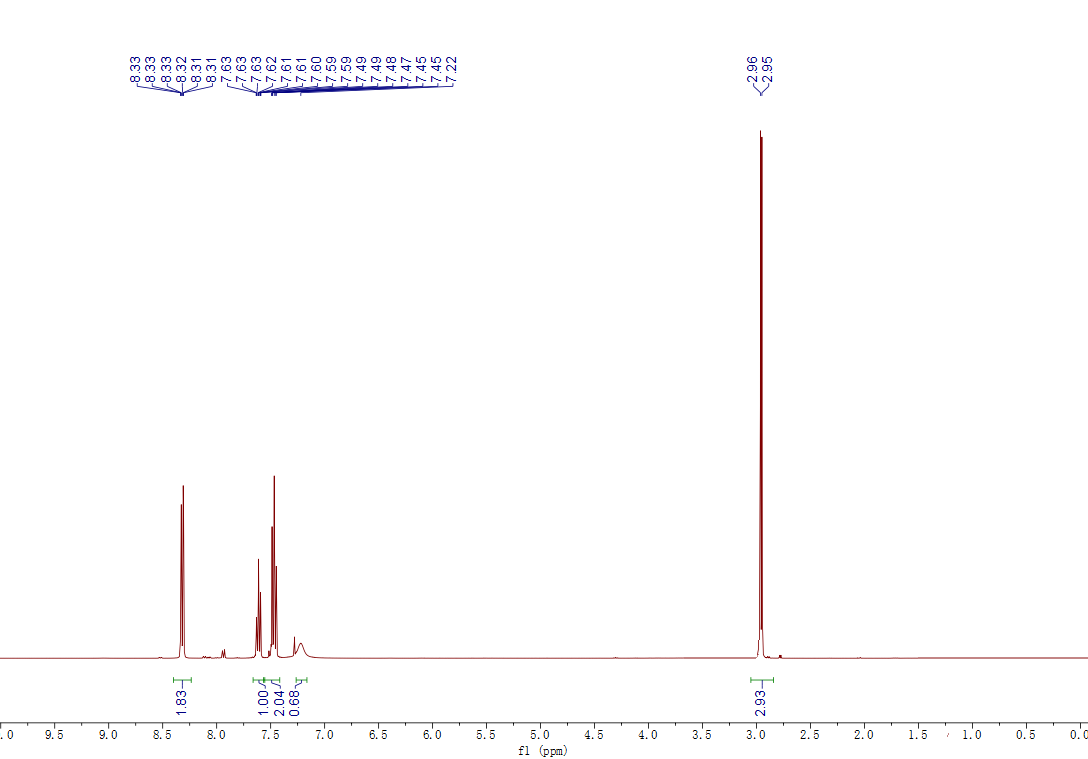


**3a**


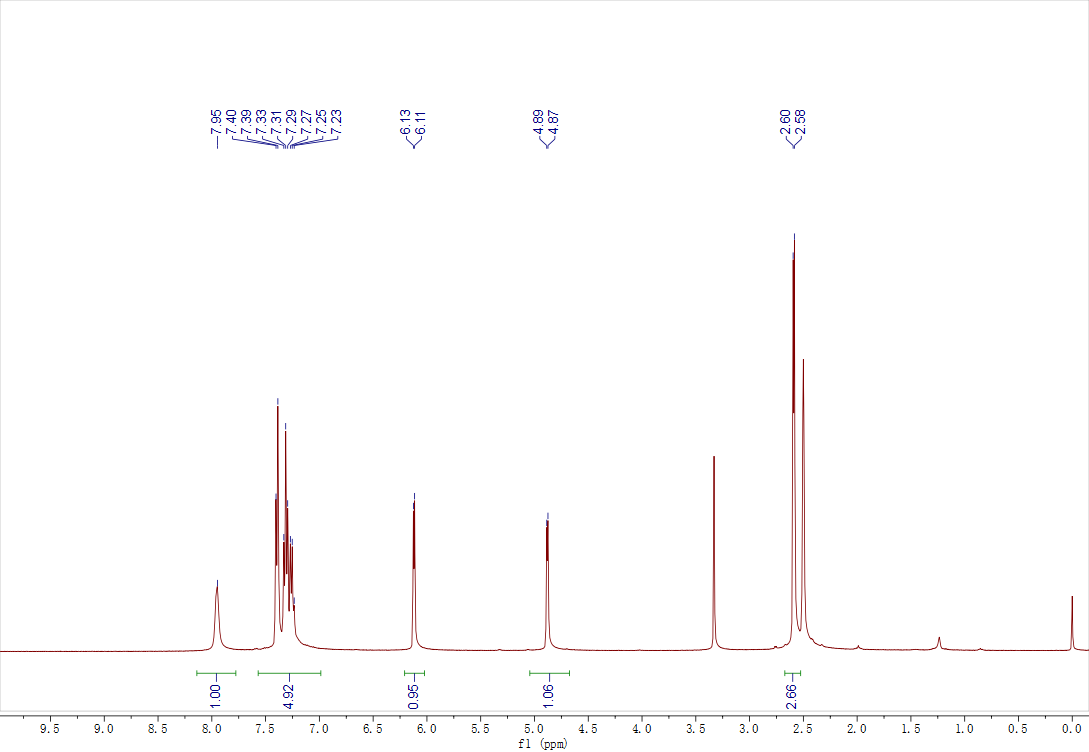


**3b**

**Figure S16**. The ^1^H NMR spectrum of **3a** and **3b**. **3a** ^1^H NMR (400 MHz, Chloroform-*d*) δ 8.32 (dt, *J* = 8.6, 1.5 Hz, 2H), 7.67 – 7.55 (m, 1H), 7.57 – 7.40 (m, 2H), 2.96 (d, *J* = 5.2 Hz, 3H). **3b** ^1^H NMR (400 MHz, DMSO-*d*_6_) δ 7.95 (q, *J* = 5.1 Hz, 1H), 7.58 – 6.95 (m, 5H), 6.12 (d, *J* = 4.5 Hz, 1H), 4.88 (d, *J* = 4.5 Hz, 1H), 2.59 (d, *J* = 4.7 Hz, 3H).


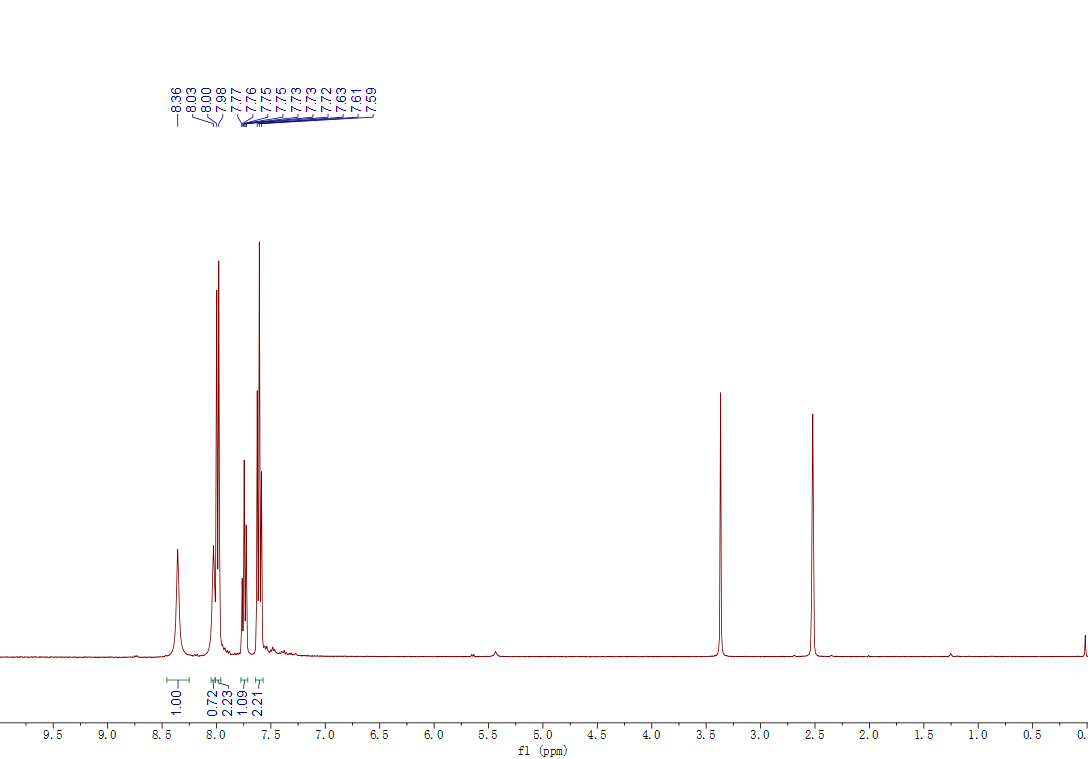


**4a**


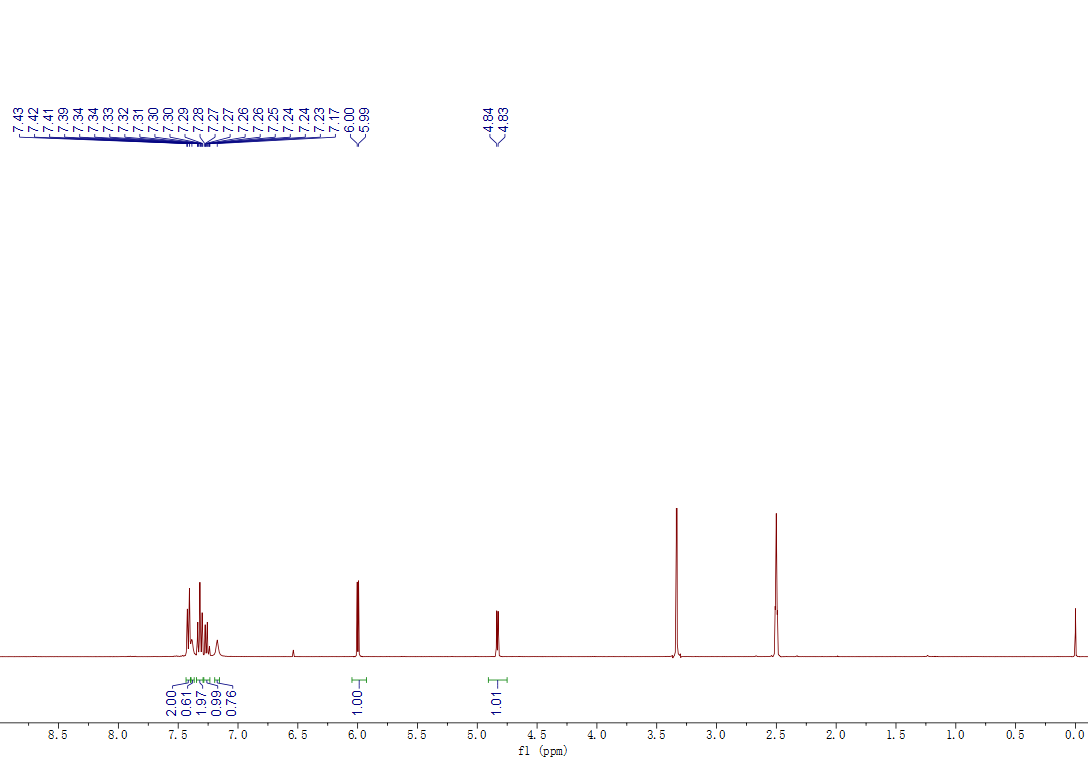


**4b**

**Figure S17**. The ^1^H NMR spectrum of **4a** and **4b**. **4a** ^1^H NMR (400 MHz, DMSO-*d*_6_) δ 8.36 (s, 1H), 8.03 (s, 1H), 7.99 (d, *J* = 7.2 Hz, 2H), 7.78 – 7.71 (m, 1H), 7.61 (t, *J* = 7.7 Hz, 2H). **4b** ^1^H NMR (400 MHz, DMSO-*d*_6_) δ 7.45 – 7.38 (m, 3H), 7.39 (s, 1H), 7.36 – 7.27 (m, 2H), 7.29 – 7.23 (m, 1H), 7.17 (s, 1H), 6.00 (d, *J* = 4.7 Hz, 1H), 4.83 (d, *J* = 4.7 Hz, 1H).


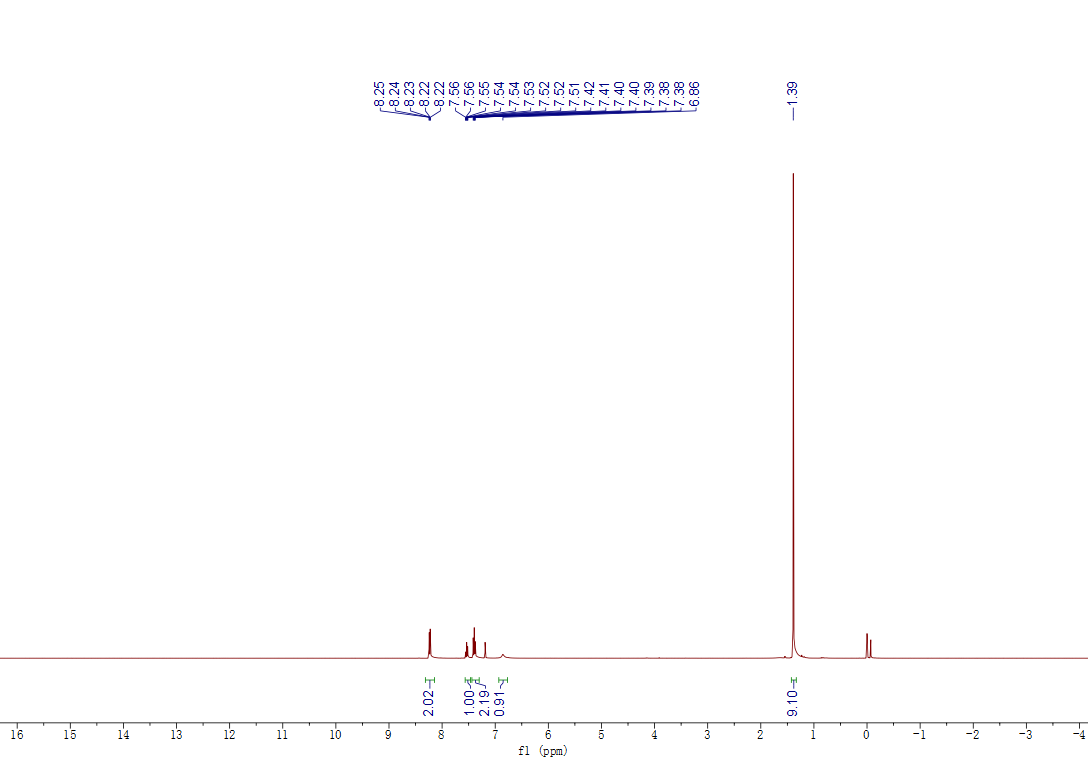


**5a**


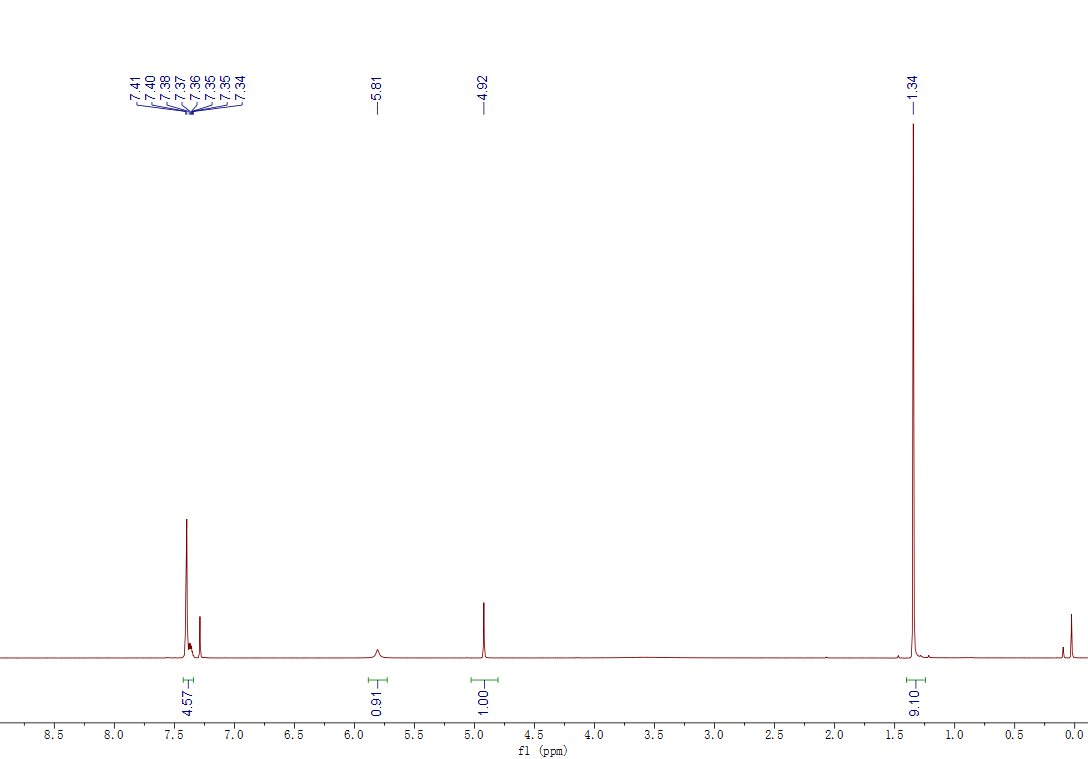


**5b**

**Figure S18**. The ^1^H NMR spectrum of **5a** and **5b**. **5a** ^1^H NMR (400 MHz, Chloroform-*d*) δ 8.23 (dd, *J* = 8.4, 1.4 Hz, 2H), 7.57 – 7.46 (m, 1H), 7.40 (dd, *J* = 8.4, 7.2 Hz, 2H), 6.86 (s, 1H), 1.39 (s, 9H).

**5b** ^1^H NMR (500 MHz, Chloroform-*d*) δ 7.43 – 7.32 (m, 5H), 5.81 (s, 1H), 4.92 (s, 1H), 1.34 (s, 9H).


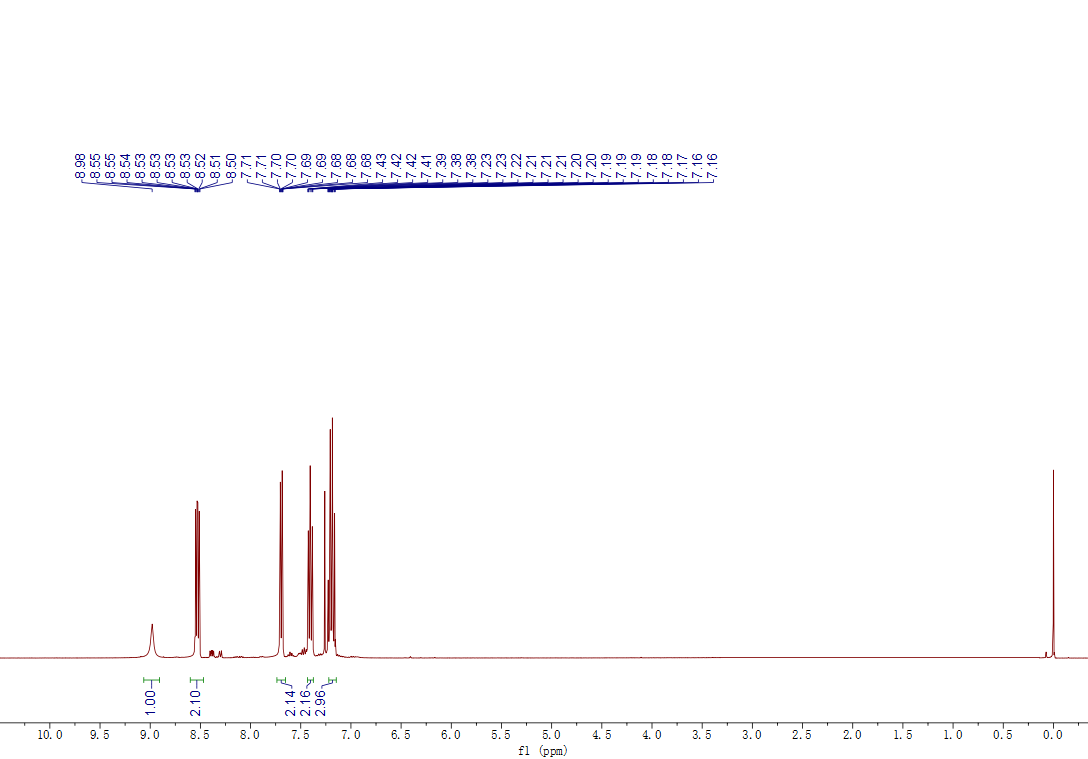


**6a**


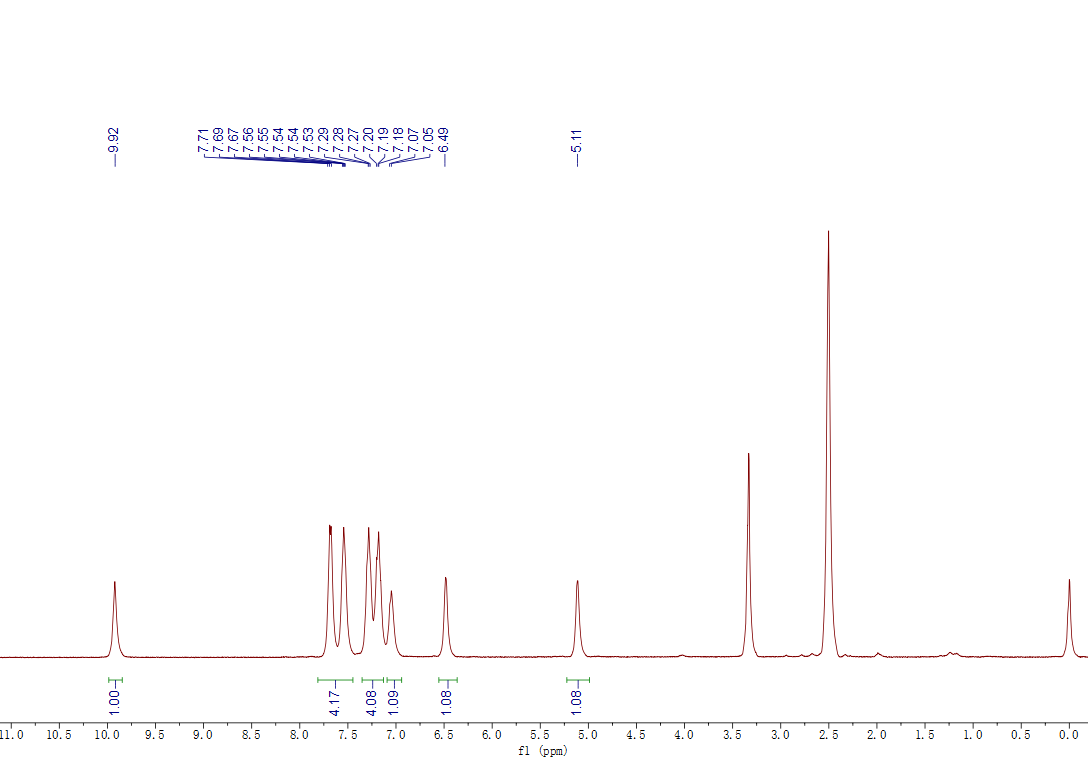


**6b**

**Figure S19**. The ^1^H NMR spectrum of **6a** and **6b**. **6a** ^1^H NMR (400 MHz, Chloroform-*d*) δ 8.98 (s, 1H), 8.60 – 8.47 (m, 2H), 7.74 – 7.65 (m, 2H), 7.45 – 7.36 (m, 2H), 7.19 (ddd, *J* = 9.0, 7.9, 2.0 Hz, 3H).

**6b** ^1^H NMR (400 MHz, DMSO-*d*_6_) δ 9.92 (s, 1H), 7.81 – 7.45 (m, 4H), 7.35 – 7.13 (m, 4H), 7.06 (d, *J* = 7.1 Hz, 1H), 6.49 (s, 1H), 5.11 (s, 1H).


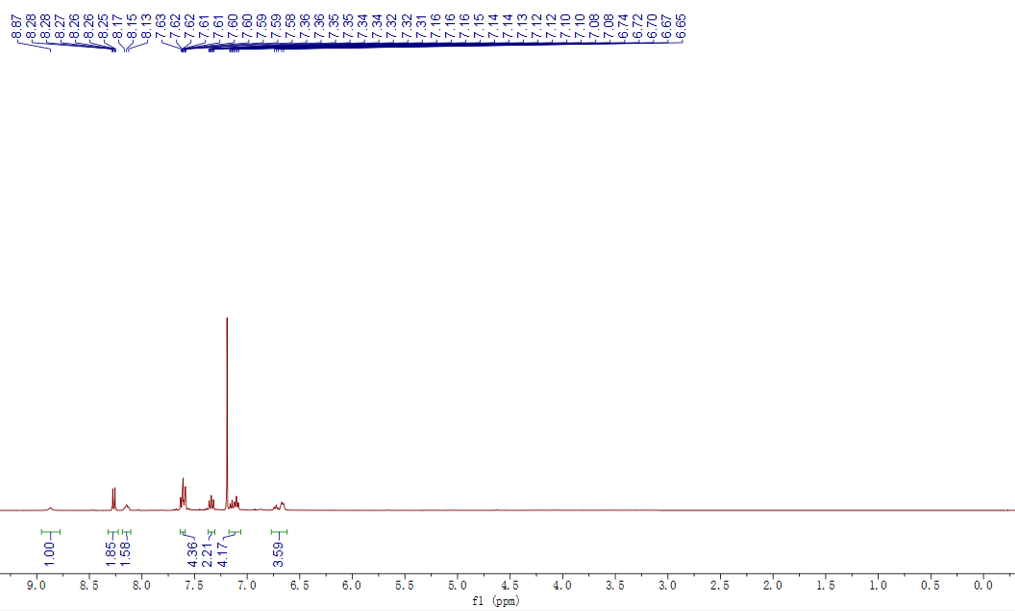

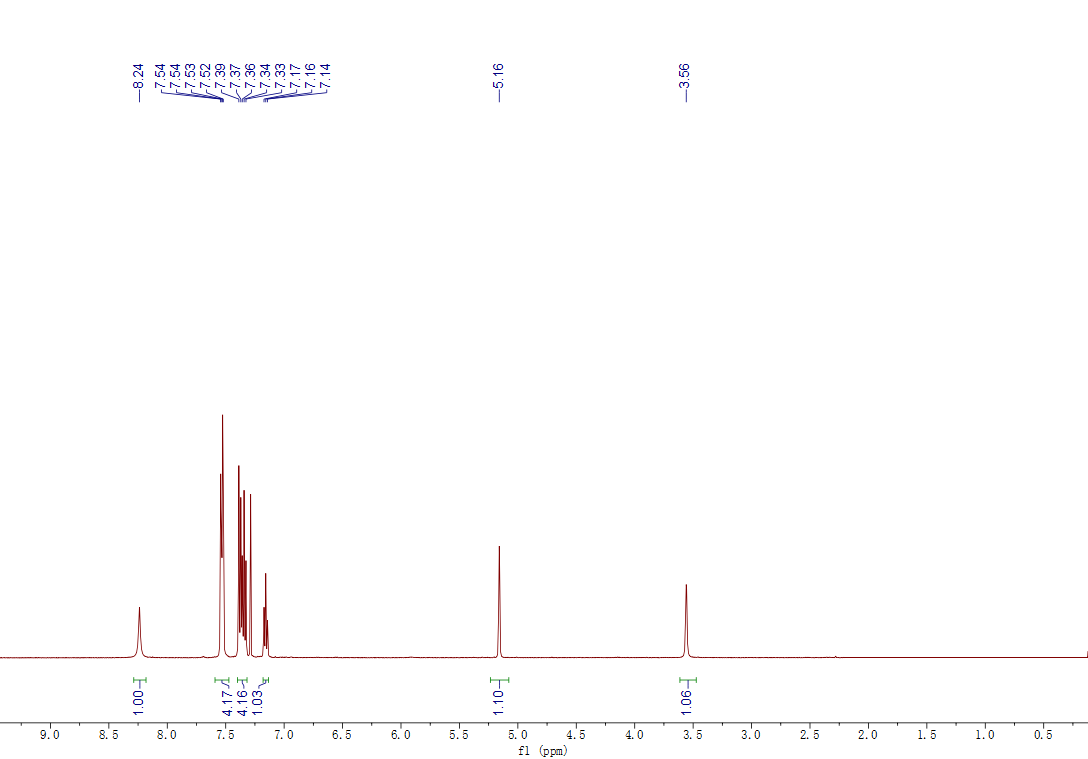


**7b**

**7a**

**Figure S20**. The ^1^H NMR spectrum of **7a** and **7b**. **7a** ^1^H NMR (400 MHz, Chloroform-*d*) δ 8.87 (s, 1H), 8.32 – 8.22 (m, 2H), 8.15 (t, *J* = 7.8 Hz, 2H), 7.65 – 7.57 (m, 5H), 7.34 (ddd, *J* = 8.5, 5.8, 2.0 Hz, 2H), 7.17 – 7.06 (m, 4H), 6.77 – 6.62 (m, 4H). **7b** ^1^H NMR (500 MHz, Chloroform-*d*) δ 8.24 (s, 1H), 7.53 (dd, *J* = 8.8, 2.4 Hz, 4H), 7.41 – 7.31 (m, 4H), 7.16 (t, *J* = 7.5 Hz, 1H), 5.16 (s, 1H), 3.56 (s, 1H).


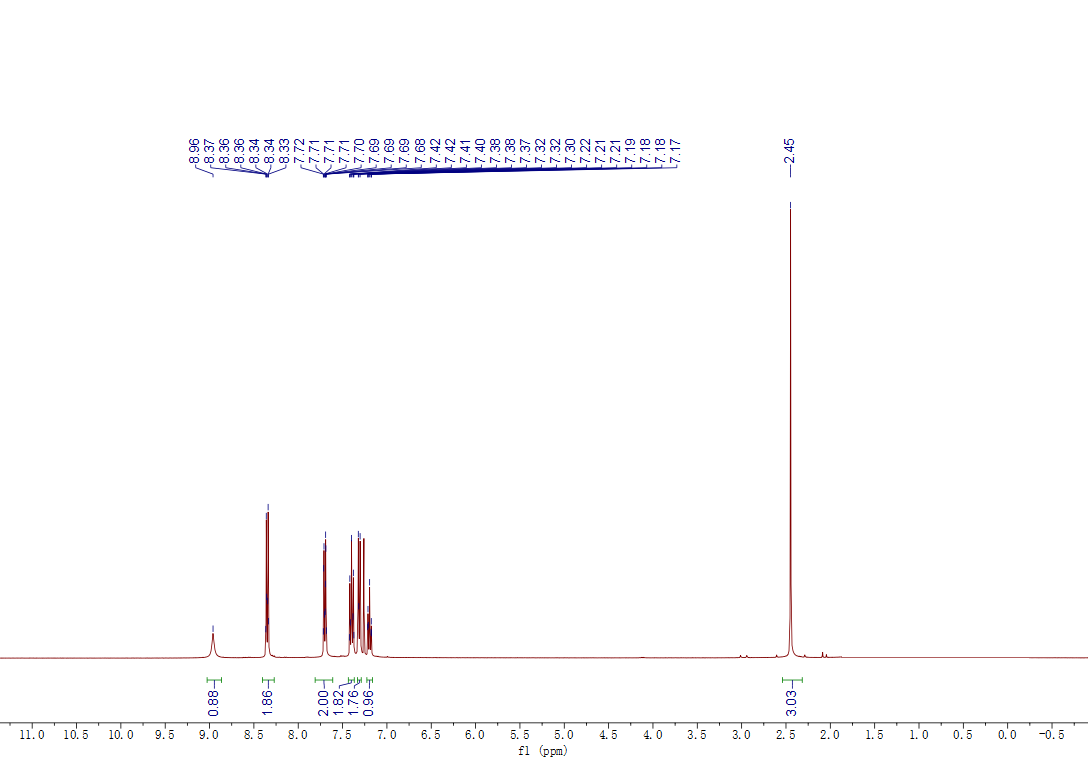


**8a**


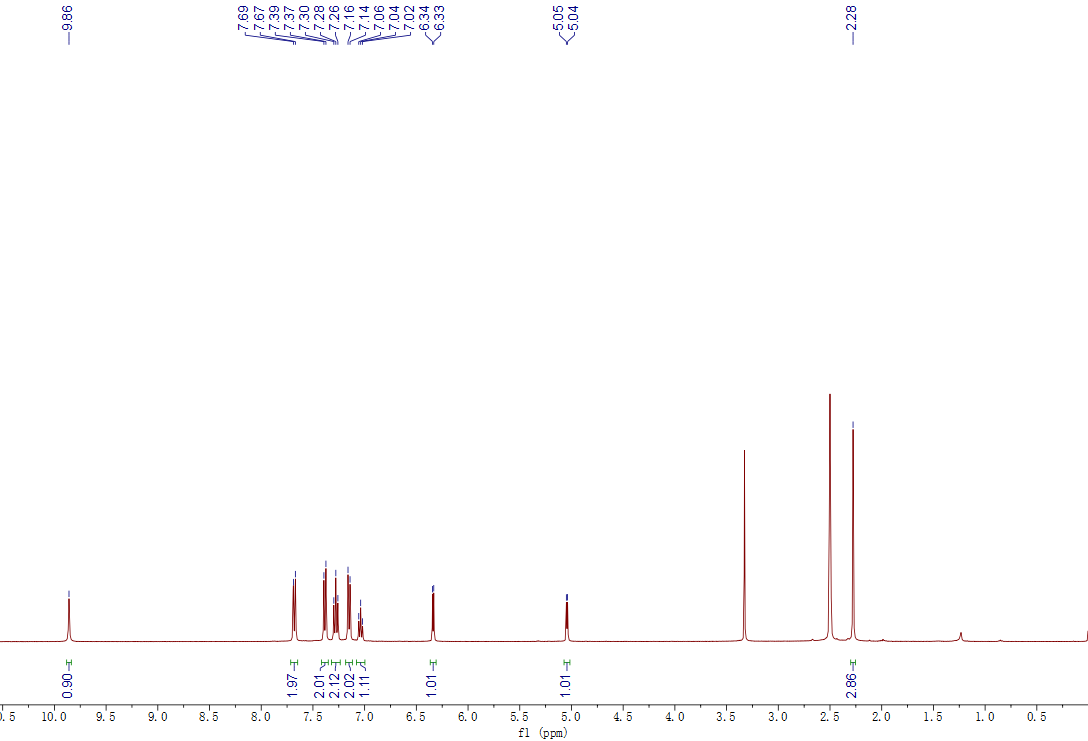


**8b**

**Figure S21**. The ^1^H NMR spectrum of **8a** and **8b**. **8a** ^1^H NMR (400 MHz, Chloroform-*d*) δ 8.96 (s, 1H), 8.40 – 8.27 (m, 2H), 7.81 – 7.61 (m, 2H), 7.45 – 7.35 (m, 2H), 7.31 (d, *J* = 8.1 Hz, 2H), 7.24 – 7.15 (m, 1H), 2.45 (s, 3H). **8b** ^1^H NMR (400 MHz, DMSO-*d*_6_) δ 9.86 (s, 1H), 7.68 (d, *J* = 7.6 Hz, 2H), 7.38 (d, *J* = 7.9 Hz, 2H), 7.28 (t, *J* = 7.9 Hz, 2H), 7.15 (d, *J* = 7.8 Hz, 2H), 7.04 (t, *J* = 7.4 Hz, 1H), 6.34 (d, *J* = 4.7 Hz, 1H), 5.04 (d, *J* = 4.7 Hz, 1H), 2.28 (s, 3H).


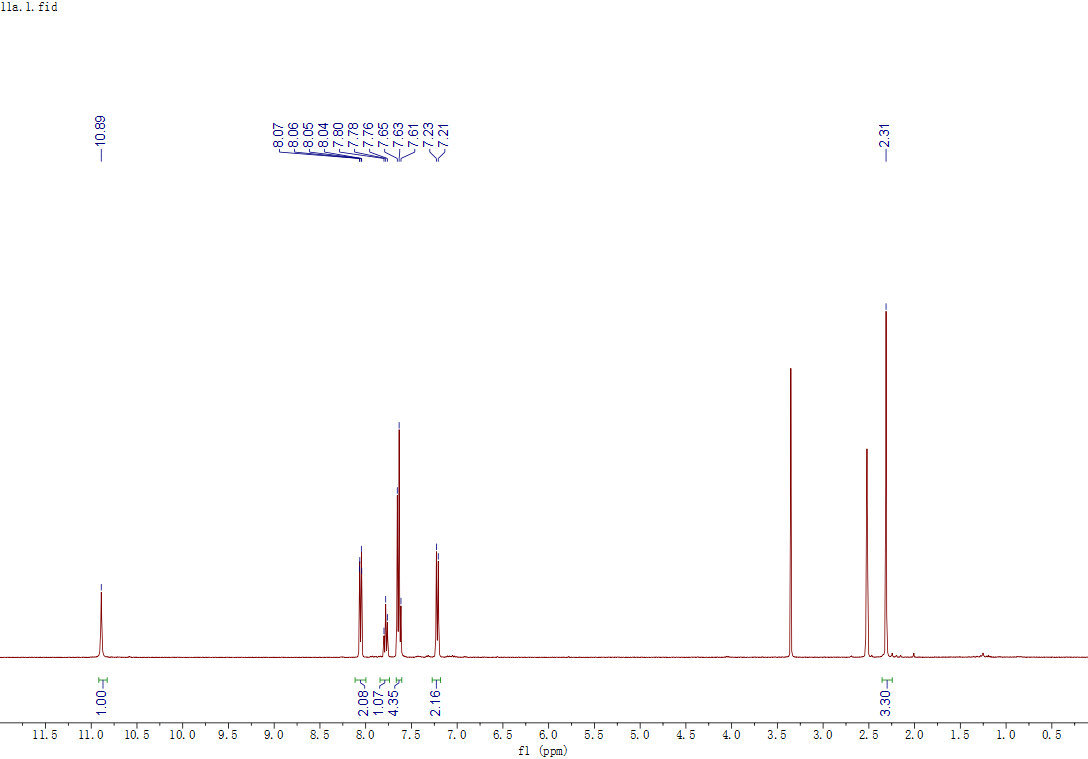


**9a**


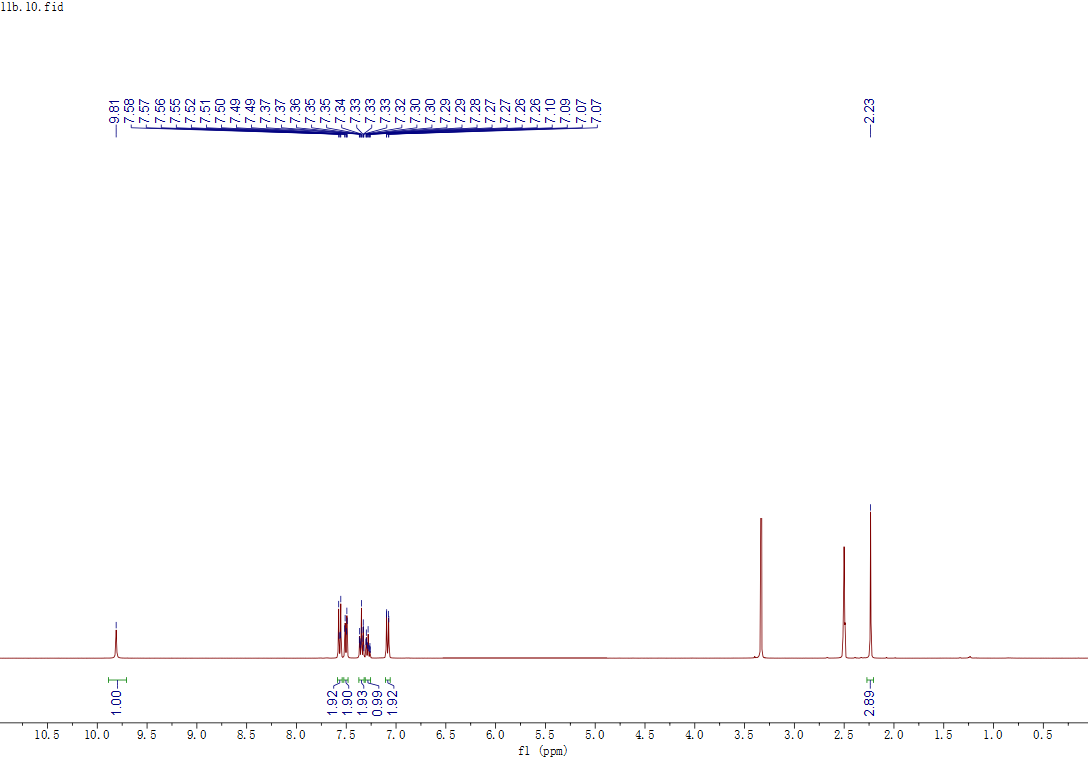


**9b**

**Figure S22**. The ^1^H NMR spectrum of **9a** and **9b**. **9a** ^1^H NMR (400 MHz, DMSO-*d*_6_) δ 10.89 (s, 1H), 8.12 – 8.00 (m, 2H), 7.78 (t, *J* = 7.4 Hz, 1H), 7.63 (t, *J* = 8.0 Hz, 4H), 7.22 (d, *J* = 8.1 Hz, 2H). **9b** ^1^H NMR (400 MHz, DMSO-*d*_6_) δ 9.81 (s, 1H), 7.60 – 7.53 (m, 2H), 7.54 – 7.46 (m, 2H), 7.37 – 7.32 (m, 2H), 7.31 – 7.26 (m, 1H), 7.12 – 7.04 (m, 2H), 2.23 (s, 3H).


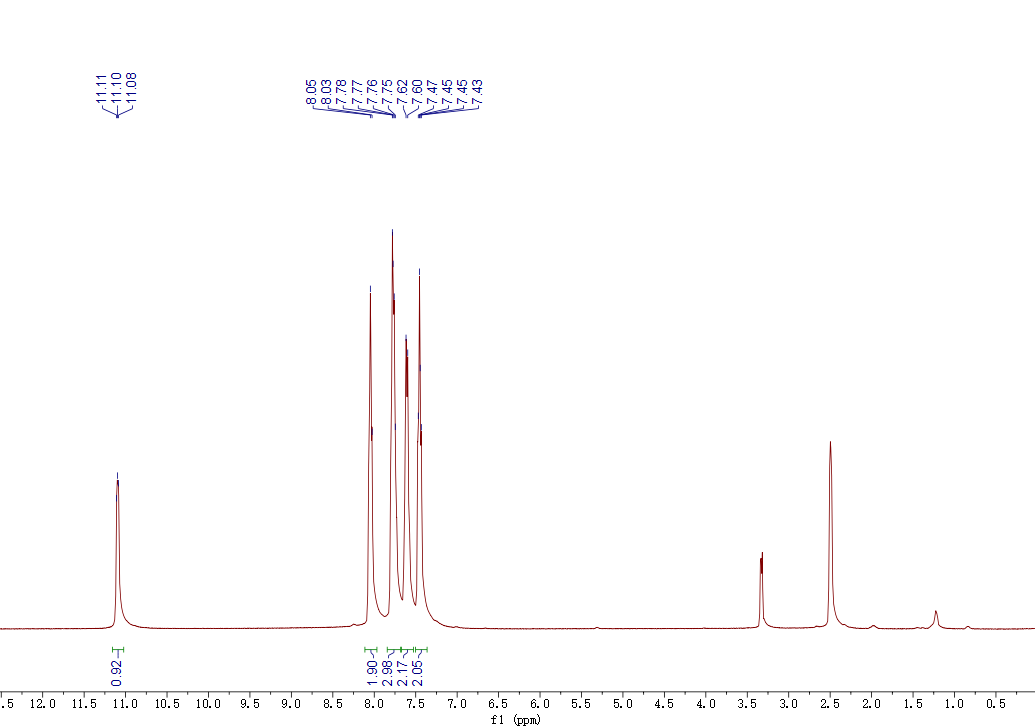


**10a**

.
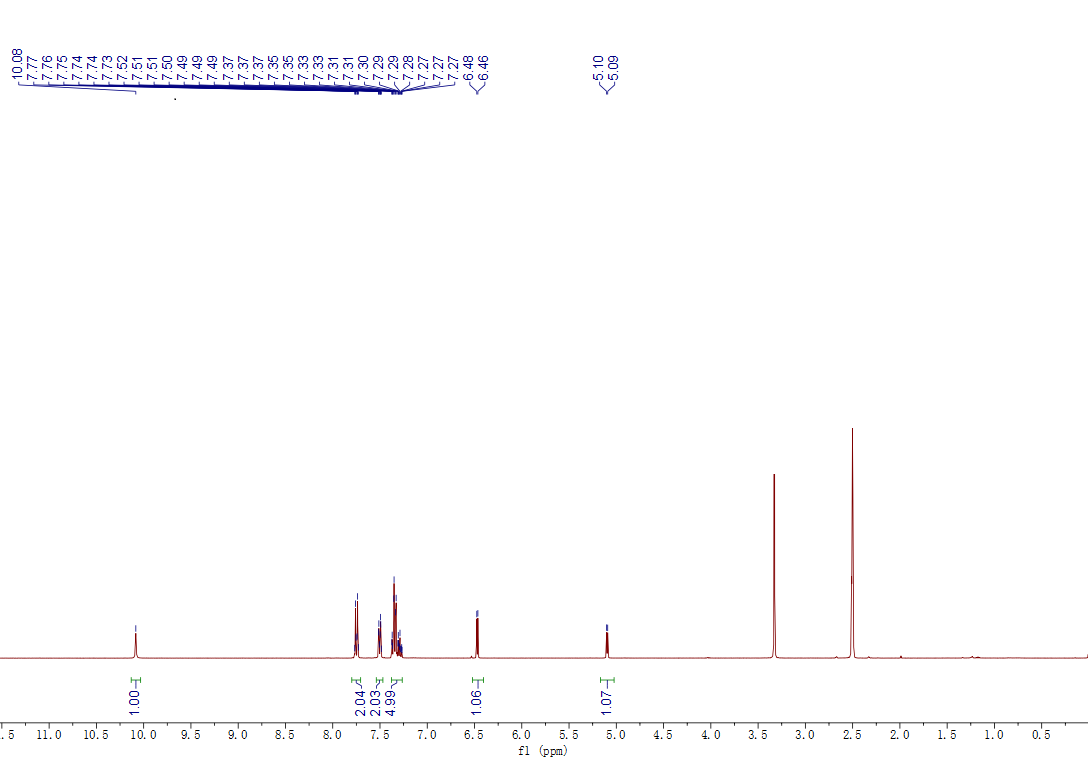


**10b**

**Figure S23**. The ^1^H NMR spectrum of **10a** and **10b**. **10a** ^1^H NMR (400 MHz, DMSO-*d*_6_) δ 11.16 – 11.02 (m, 1H), 8.04 (d, *J* = 8.2 Hz, 2H), 7.76 (dd, *J* = 9.9, 4.4 Hz, 3H), 7.61 (d, *J* = 8.2 Hz, 2H), 7.45 (t, *J* = 7.4 Hz, 2H). **10b** ^1^H NMR (400 MHz, DMSO-*d*_6_) δ 10.08 (s, 1H), 7.80 – 7.70 (m, 2H), 7.54 – 7.47 (m, 2H), 7.40 – 7.24 (m, 5H), 6.47 (d, *J* = 4.6 Hz, 1H), 5.10 (d, *J* = 4.6 Hz, 1H).
